# Mesenchymal Stem Cells Attenuate Peritoneal Injury through Secretion of TSG-6

**DOI:** 10.1371/journal.pone.0043768

**Published:** 2012-08-17

**Authors:** Nan Wang, Qinggang Li, Li Zhang, Hongli Lin, Jie Hu, Diangeng Li, Suozhu Shi, Shaoyuan Cui, Jianhui Zhou, Jiayao Ji, Jiajia Wan, Guangyan Cai, Xiangmei Chen

**Affiliations:** 1 State Key Laboratory of Kidney Diseases, Department of Nephrology, PLA General Hospital and Military Medical Postgraduate College, Beijing, China; 2 Medical College, NanKai University, Tianjin, China; 3 Department of Nephrology, the First Affiliated Hospital of Dalian Medical University, Liaoning, China; Universidade de Sao Paulo, Brazil

## Abstract

**Background:**

Mesothelial cell injury plays an important role in peritoneal fibrosis. Present clinical therapies aimed at alleviating peritoneal fibrosis have been largely inadequate. Mesenchymal stem cells (MSCs) are efficient for repairing injuries and reducing fibrosis. This study was designed to investigate the effects of MSCs on injured mesothelial cells and peritoneal fibrosis.

**Methodology/Principal Findings:**

Rat bone marrow-derived MSCs (5 ×10^6^) were injected into Sprague-Dawley (SD) rats via tail vein 24 h after peritoneal scraping. Distinct reductions in adhesion formation; infiltration of neutrophils, macrophage cells; number of fibroblasts; and level of transforming growth factor (TGF)-β1 were found in MSCs-treated rats. The proliferation and repair of peritoneal mesothelial cells in MSCs-treated rats were stimulated. Mechanically injured mesothelial cells co-cultured with MSCs in transwells showed distinct increases in migration and proliferation. *In vivo* imaging showed that MSCs injected intravenously mainly accumulated in the lungs which persisted for at least seven days. No apparent MSCs were observed in the injured peritoneum even when MSCs were injected intraperitoneally. The injection of serum-starved MSCs-conditioned medium (CM) intravenously reduced adhesions similar to MSCs. Antibody based protein array of MSCs-CM showed that the releasing of TNFα-stimulating gene (TSG)-6 increased most dramatically. Promotion of mesothelial cell repair and reduction of peritoneal adhesion were produced by the administration of recombinant mouse (rm) TSG-6, and were weakened by TSG-6-RNA interfering.

**Conclusions/Significance:**

Collectively, these results indicate that MSCs may attenuate peritoneal injury by repairing mesothelial cells, reducing inflammation and fibrosis. Rather than the engraftment, the secretion of TSG-6 by MSCs makes a major contribution to the therapeutic benefits of MSCs.

## Introduction

The mesothelial layer is important in the maintenance of peritoneal homeostasis, including transport and movement of fluid and particulate material; the synthesis of pro-/anti-inflammatory and immunomodulatory cytokines, growth factors, and extracellular matrix (ECM); and the control of fibrinolysis [1]. Tissue repair commences after mesothelial injury. This process is characterized by remesothelialization of the injured area, neovascularization, and fibrosis of the submesothelial ECM with an influx of neutrophils, lymphocytes, and macrophages [2]. Denudation of the mesothelial cells is observed with increasing time in end-stage renal disease (ESRD) patients with peritoneal dialysis (PD) [3], and the loss of mesothelial cells correlates with the extent of peritoneal fibrosis and ultrafiltration failure [4], [5]. Besides, mesothelial cell denudation and damage also play an important role in the formation of postoperative peritoneal adhesions [6], [7]. No satisfactory solution has proved to ameliorate peritoneal fibrosis efficiently. Recent studies have shown that the recovery of surgically damaged mesothelium reduces the peritoneal adhesions and fibrosis, resulting in improved structural repair and function of peritoneum [6], [8]. Therefore, promoting the mesothelial recovery may be a novel therapeutic target to reduce peritoneal fibrosis.

Recent reports have demonstrated the capacity of mesenchymal stem cells (MSCs) to repair tissue injuries [9]. Moreover, animal models have demonstrated that MSCs decrease fibrosis in the heart [10], lung [11], liver [12] and kidney [13]. MSCs transplantation is considered safe and has been widely tested in clinical trials with encouraging results [14]. MSCs possess many peculiarities, including the multilineage potential [15], high immune privilege, the immunomodulatory properties and the easy self-renewal *in vitro* [14]. However, the mechanisms by which MSCs exert their beneficial effects remain controversial. Many researchers believe that the effect is mediated by an increase in angiogenic [16], mitogenic [17], cytoprotective, anti-inflammatory, anti-apoptotic, immunosuppressive and anti-fibrogenic factors [18], [19], as well as differentiation into specific cell types [20]. To our limited knowledge, there has been no report relevent to the effects of MSCs on peritoneal injury induced by non-infectious factors. TNFα-stimulating gene (TSG)-6, a therapeutic protein produced by MSCs in response to injury signals, can limit destruction of the tissue by excessive inﬂammatory response to a sterile injury [21]. Research find that MSCs may be activated by the inflammatory microenvironment of the peritoneal cavity to secrete TSG-6, and attenuate peritonitis induced by zymosan [22].

To investigate the effects of MSCs on injured mesothelial cells and peritoneal fibrosis, we established a quantifiable acute peritoneal adhesion rat model by mechanically scraping mesothelial layers *in vivo* [8], [23], [24], and a mechanically scraped injury model of primary rat peritoneal mesothelial cells (RPMCs) *in vitro* [25]. We demonstrate that MSCs reduce acute peritoneal adhesion/fibrosis induced b*y* mesothelial cell injury without engraftment in the peritoneum and primarily through secreting TSG-6. We also demonstrate that similar therapeutic effects were produced by the administration of TSG-6.

## Methods

### Ethics Statement

This study was approved by the Ethics Committee of The General Hospital of the People's Liberation Army (Permit Number: 2010-X-3–28) with animal care performed strictly according to established institutional guidelines. All surgery was performed under pentobarbital anesthesia, and all efforts were made to minimize suffering.

**Figure 1:**
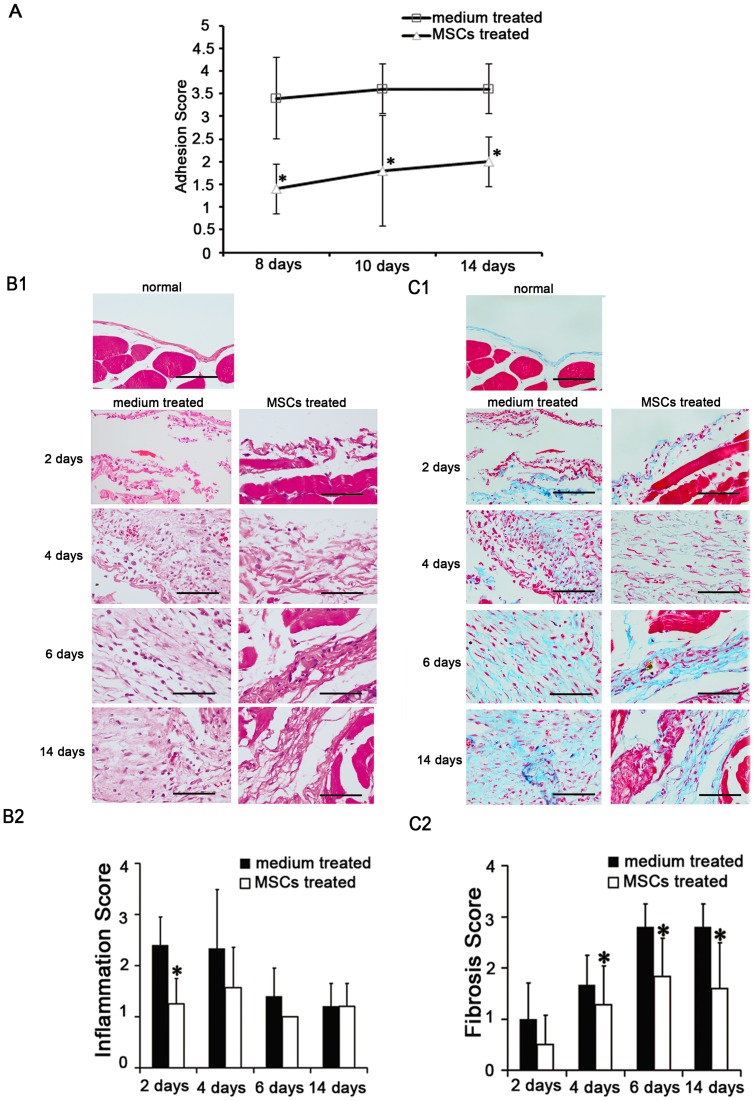
Effects of mesenchymal stem cells (MSCs) on the severity and the histological changes of acute peritoneal adhesions. (A). The MSCs treated group had lower adhesion scores. The size and severity of peritoneal adhesion were evaluated macroscopically by an independent observer on a scale of 0–4 (0, 0%; 1, <25%; 2, 25–49%; 3, 50–74%; and 4, 75–100% adhesions). * compared with medium treated group, *p* <0.05, n  = 6, respectively. (B). HE staining revealed the changes of inflammation in acute peritoneal adhesions. (B1). Peritoneal inflammations after scraping were reduced by injecting MSCs. Magnification  = ×400. (B2). The scores of peritoneal inflammation days 2 after scraping were reduced by injecting MSCs. * compared with medium treated group, *p* <0.05, n  = 6, respectively. (C). Masson's trichrome staining revealed the changes of fibrosis in acute peritoneal adhesions. (C1). Peritoneal fibroses after scraping were reduced by injecting MSCs. Magnification  = ×400. (C2). The scores of peritoneal fibrosis days 4, 6, 14 after scraping were reduced by injecting MSCs. * compared with medium treated group, *p* <0.05, n  = 6, respectively. The sections were evaluated from five randomly selected fields under a magnification of ×100 by an independent pathologist. The extents of inflammation (HE staining) and fibrosis (masson's trichrome staining) were scored as 0 (negative), 1 (weak), 2 (medium), or 3 (intensive), respectively.

**Figure 2:**
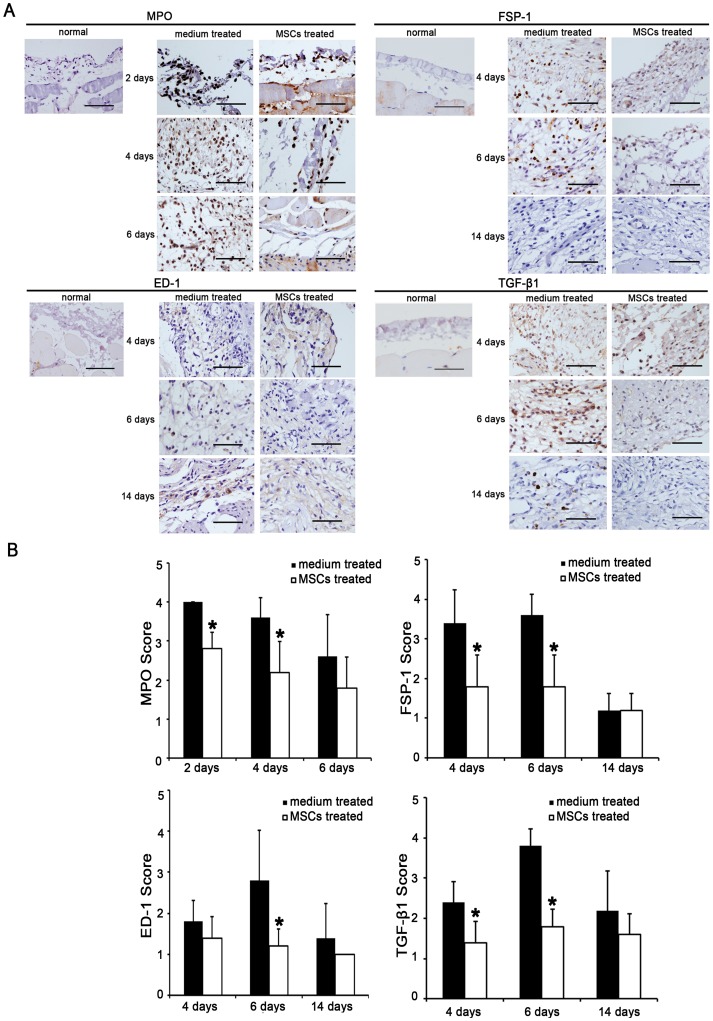
Effects of mesenchymal stem cells (MSCs) on the inflammation and fibrosis of acute peritoneal adhesions. (A). Immunohistochemical evaluation revealed that the numbers of fibroblasts (FSP-1), neutrophils (MPO), and macrophage cells (ED-1), and the level of transforming growth factor (TGF)-β1 during the active phase in acute peritoneal adhesions were decreased by injecting MSCs. Magnification  = ×400. (B). The immunohistochemical scores of MPO days 2, 4, ED-1 days 6, FSP-1 and TGF-β1 days 4, 6 after scraping were reduced by injecting MSCs. * compared with medium treated group, *p* <0.05, n  = 6, respectively. Sections were evaluated from five randomly selected fields under a magnification of ×100 by an independent pathologist. The extent of staining was scored as 0 (0%), 1 (1–20%), 2 (21–50%), 3 (51–80%), and 4 (81–100%), indicating the percentage of positive staining in the adhesion tissue.

### Acute peritoneal adhesion rat models

Scraping-induced peritoneal adhesions were created in healthy male Sprague-Dawley (SD) rats weighing 200–250 g each. All animals were obtained from the Experimental Animal Center of the Academy of Military Medical Sciences (Beijing, China) and housed at a constant room temperature with a 12 h light/dark cycle. Standard rodent chow and water were provided *ad libitum*. The animals were acclimated for seven days before initiating the experiment. All surgical procedures were conducted by a single surgeon under aseptic conditions in the Laboratory Animal Unit. Rats were anesthetized with a 2% pentobarbital (30 mg/kg) intraperitoneal injection. Briefly, a 2-cm vertical midline incision was made through the abdominal wall and peritoneum. The dorsal and ventral surfaces of the cecum were scraped with dry gauze 20 times over an area of 2×2 cm^2^ until petechial bleeding occurred. The cecum was then placed back into the abdominal cavity in its natural position. The parietal peritoneum lateral to the midline incision was also scraped 20 times until petechial bleeding occurred. The abdominal incision was closed in two layers with 4/0 silk sutures [8]. After the operation, the rat was kept in a single cage, maintained on a regular diet, and monitored daily for postoperative complications.

**Figure 3:**
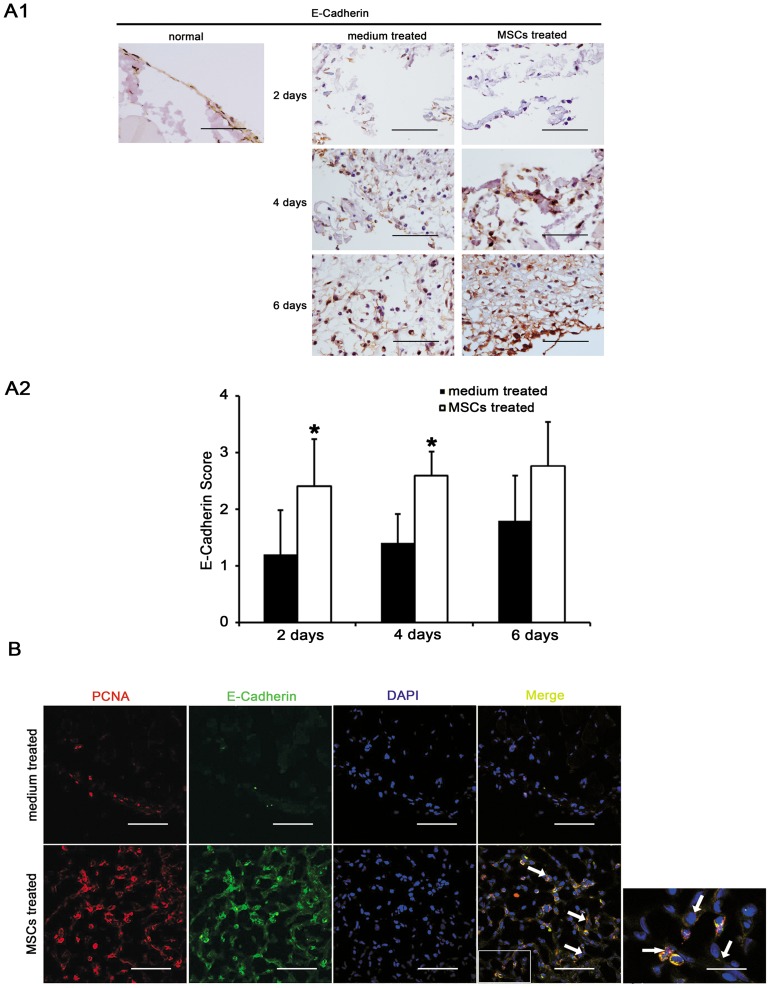
Effects of mesenchymal stem cells (MSCs) on the repair of peritoneal mesothelial cells. (A). Immunohistochemical evaluation of peritoneal mesothelial cells (E-Cadherin) in acute peritoneal adhesions. (A1). The number of mesothelial cells after peritoneal scraping was increased by injecting MSCs. Magnification  = ×400. (A2). The immunohistochemical scores of E-Cadherin days 2, 4 after scraping were increased by injecting MSCs. * compared with medium treated group, *p* <0.05, n  = 6, respectively. Sections were evaluated from five randomly selected fields under a magnification of ×100 by an independent pathologist. The extent of staining was scored as 0 (0%), 1 (1–20%), 2 (21–50%), 3 (51–80%), and 4 (81–100%), indicating the percentage of positive staining in the adhesion tissue. (B). Immunofluorescence evaluation of injured peritoneum days 4 after scraping using antibodies to E-Cadherin (green) and PCNA (red). The nucleus was counterstained with DAPI (blue). Dual-stained cells (indicated as arrows) were predominantly expressed in the MSCs treated group. Magnification  = ×600 and ×1800, when necessary.

### MSCs culture

SD rat bone marrow-derived MSCs/green fluorescent protein (GFP) were obtained commercially (Cyagen Biosciences, Sunnyvale, CA). The culture process was initiated according to the manufacturer's instructions. MSCs were placed into 25 cm^2^ culture flasks (Corning, Corning, NY) and cultured with MSCs growth medium (Cyagen Biosciences, Sunnyvale, CA) at 37°C under 5% CO_2_ and 90% humidity. The medium was changed every three days. The sixth-eighth passage MSCs were used for experiments. According to previously described methods [26], fluorescence activated cell sorting (FACS) analysis (Beckman Coulter, Indianapolis) was performed to examine the representative markers characteristic of MSCs (CD45, CD90 (BD Biosciences Pharmingen, San Diego, California); CD11a, CD54 (AbD Serotec, Oxford, UK)), and the multilineage differentiation of MSCs was examined under adipogenic and osteogeneic differentiation conditions, prior to using for *in vitro* and *in viv*o experiments.

**Figure 4:**
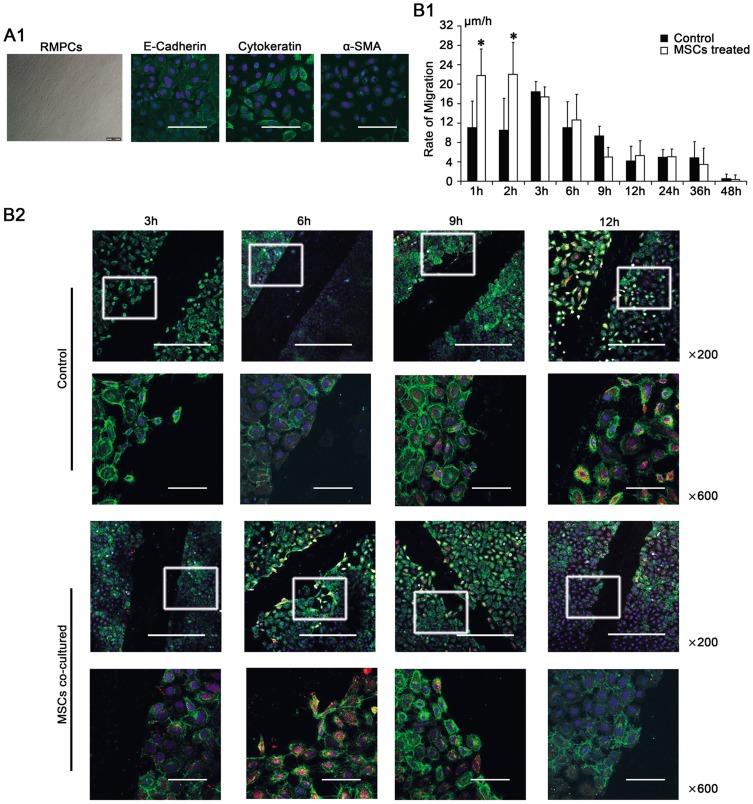
Effects of mesenchymal stem cells (MSCs) on the repair of rat peritoneal mesothelial cells (RPMCs) following mechanical injury. (A). Characteristics of primary RPMCs cultures. (A1). Photomicrograph showing the cobblestone-like appearance of the epithelioid phenotypes. Magnification  = ×100. (A2). An immunofluorescence evaluation revealed that RPMCs expressed E-Cadherin (green) and Cytokeratin (green), but only weakly expressed α-SMA (green). Nuclei were stained with DAPI (blue). Magnification  = ×600. (B). The effects of MSCs on the migratory and proliferative capacities of mechanically injured RPMCs. (B1). Microscopy of RPMCs migrating to the edge of the wound to show the migratory velocity (μm/h). During the first 2 h, injured RPMCs co-cultured with MSCs showed a greater migratory velocity. * compared with control group, *p* <0.05, n  = 3, respectively. (B2). Immunofluorescence analysis with PCNA (red) and E-Cadherin (green) revealed that the proliferation peak of injured RPMCs co-cultured with MSCs was accelerated to 6 h after scraping, whereas the proliferation peak was 12 h after scraping in control group. Nuclei were stained with DAPI (blue). Magnification  = ×200 and ×600.

### Transfection of MSCs with TSG-6 small interfering RNA (siRNA)

Fifty percent confluent MSCs were transfected with 20 nM TSG-6-small interfering (si) RNA or siRNA-negative control (NC) (Shanghai GenePharma., Shanghai, China) using INTERFERinTM (Polyplus-transfection SA, Bioparc, France). 24 h after the start of transfection, MSCs were fed with serum-free medium for 24 h (the same time point when TSG-6-siRNA MSCs were used for experiments). To confirm the knockdown of TSG-6, RNA was extracted from cells and assayed for TSG-6 by reverse-transcriptase polymerase chain reaction (RT-PCR) (TSG-6 forward primer: AGTGATGCGTCCGTCACAGCC, reverse primer: AGATGGCTAAACCGTCCAGCTAAGA, product length  = 134 bp; GAPDH forward primer: GGCATGGACTGTGGTCATGAG, reverse primer: TGCACCACCAACTGCTTAGC, product length  = 87 bp (SBS Genetech, Beijing, China)), conditioned medium (CM) was collected, concentrated and assayed for TSG-6 by Enzyme-linked immunosorbent assay (ELISA) (R&D Systems Inc., Mineapolis, MN).

**Figure 5:**
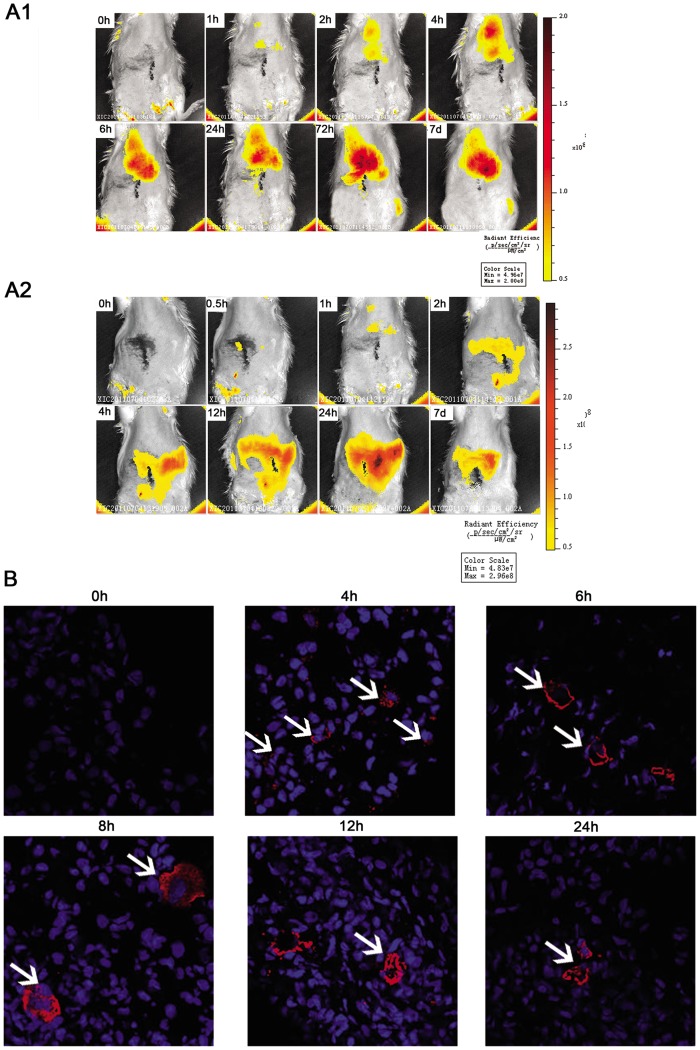
Tracking the distribution of mesenchymal stem cells (MSCs) injected into rats. (A1). *In vivo* imaging revealed that MSCs injected via tail vein first accumulated in the lungs and gradually in the liver and the spleen. (A2). *In vivo* imaging revealed that MSCs injected intraperitoneally first accumulated in the liver and gradually in the spleen. No signal was found in the injured peritoneum. (B). An immunofluorescence evaluation of green fluorescent protein (GFP) (red) detected MSCs in the lungs. Nuclei were stained with DAPI (blue). Magnification  = ×1000.

### Preparation of CM

Eighty percent confluent MSCs, TSG-6-siRNA or siRNA-NC MSCs were washed three times with phosphate buffered saline (PBS) (ZSGB-Bio, Beijing, China) and then fed with serum-free medium for 24 h [27]. The medium was collected and the cells were counted for normalization purposes. For each animal, we used medium generated by 5×10^6^ cells. After removing the cell debris by centrifugation at 2000 rpm for 10 min at 4°C, the supernatant was transferred into centrifugal filter units with 3 kDa cut-off (Millipore, Billerica, MA), concentrated 30-fold, and desalted according to the manufacturer's protocol. CM was then sterilized by filtration through a 220 nm filter (Millipore, Billerica, MA).

**Figure 6:**
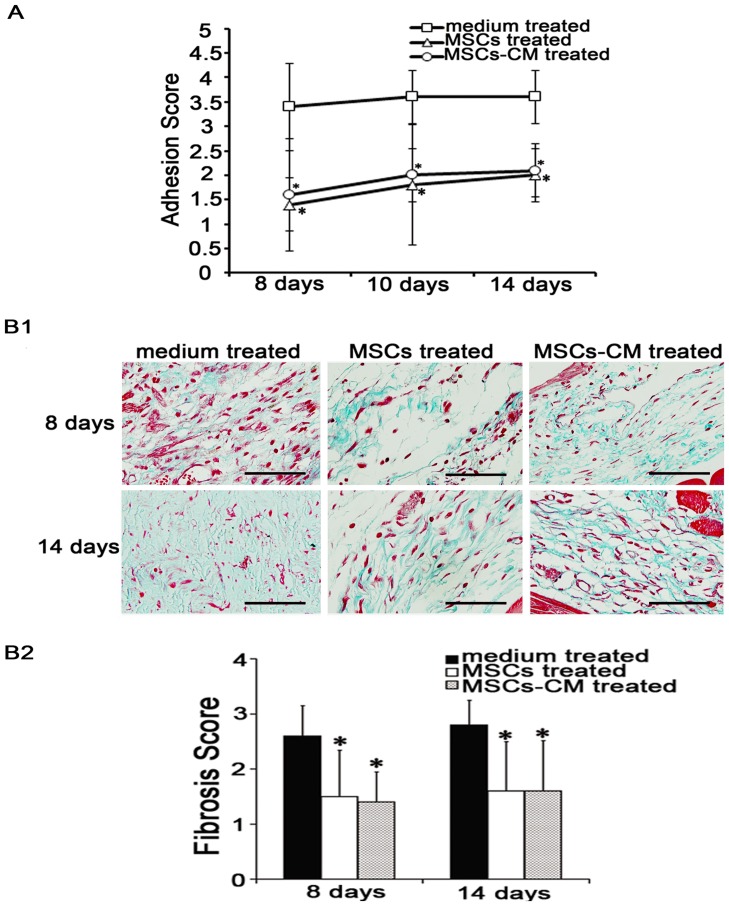
Effects of mesenchymal stem cells (MSCs)-conditioned medium (CM) on acute peritoneal adhesions. (A). The MSCs-CM treated group had lower adhesion scores days 14 after scraping. The size and severity of peritoneal adhesions were evaluated macroscopically by an independent observer on a scale of 0–4 (0, 0%; 1, <25%; 2, 25–49%; 3, 50–74%; and 4, 75–100% adhesions).* compared with medium treated group, p <0.05, n  = 6, respectively. (B). Masson's trichrome staining revealed that the changes of fibrosis in acute peritoneal adhesions. (B1). The fibrosis in the scraped peritoneum days 8, 14 after scraping was decreased by injecting MSCs-CM. Magnification  =  ×400. (B2). The scores of peritoneal fibrosis were reduced by injecting MSCs-CM, similar to MSC. * compared with medium treated group, *p* <0.05, n  = 6, respectively. The sections were evaluated from five randomly selected fields under a magnification of ×100 by an independent pathologist. The extent of fibrosis was scored as 0 (negative), 1 (weak), 2 (medium), or 3 (intensive).

### Injection of MSCs or CM

At 24 h after peritoneal scraping, the rats were allocated randomly to the MSCs or CM treated group (n  = 108) and medium treated groups (n  = 60). MSCs (5×10^6^) were injected via tail vein in a 1-ml serum-free medium. MSCs-CM, TSG-6-siRNA MSCs-CM, siRNA-NC MSCs-CM or recombinant mouse (rm) TSG-6 in serum-free medium (3.0 ng/ml, 97% homology with rat) (R&D Systems Inc., Mineapolis, MN) in a 1-ml volume was injected via tail vein on days 1, 3, and 5 after scraping. Cells were harvested and washed twice with PBS. Prior to injection, the cells were maintained at 4°C. Rat treated with serum-free medium was the negative control, and rat without peritoneal scraping was the blank control.

**Figure 7:**
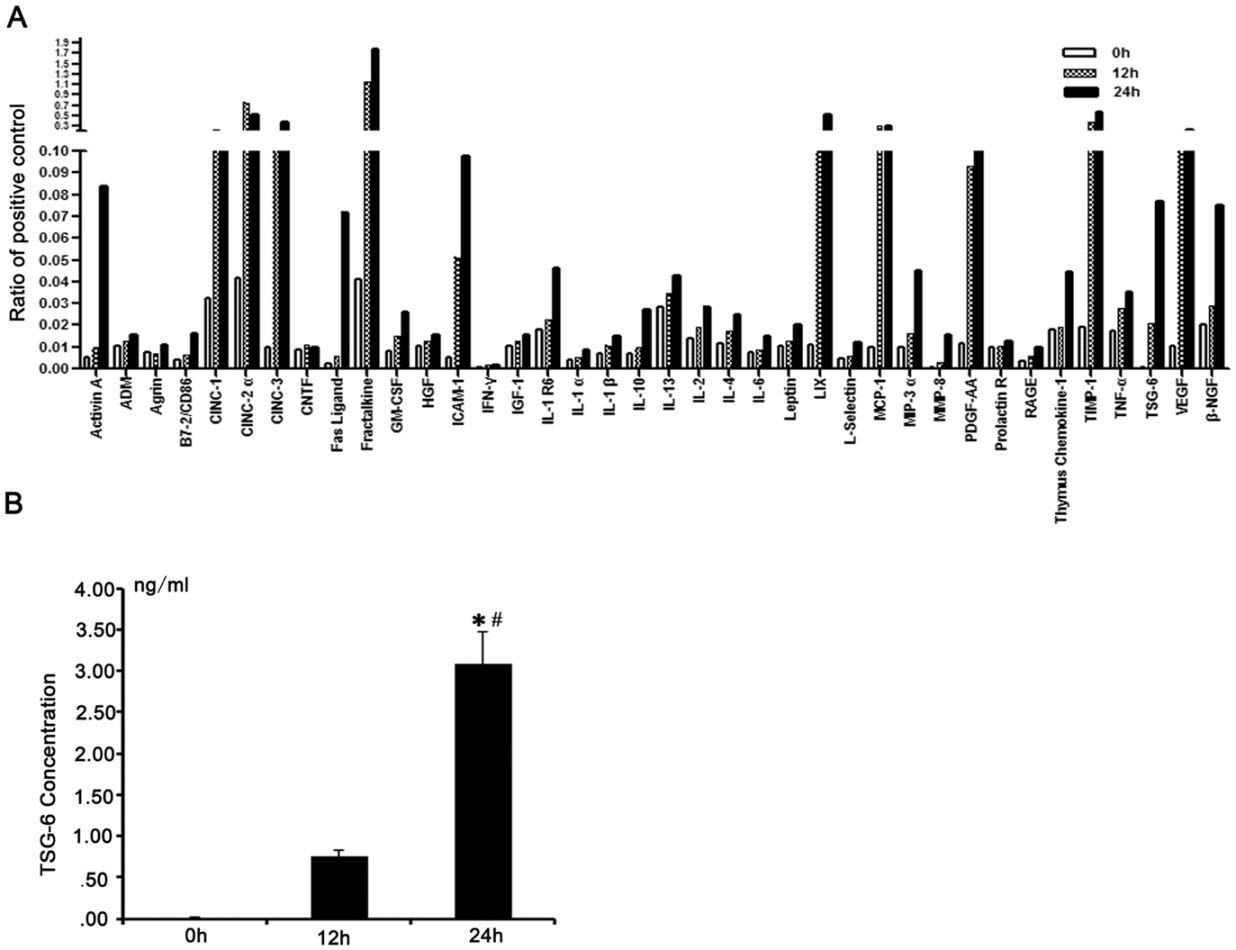
Release of TNFα-stimulating gene (TSG)-6 by mesenchymal stem cells (MSCs). (A). Cytokine profile of 30-folded serum-starved MSCs-conditioned medium (CM) within 24 h was analyzed by rat cytokine antibody array and label-based rat antibody array. Compared with 0 h MSCs-CM, wound healing cytokine TSG-6 was released abundantly by 12 h (52.7-fold) and 24 h (194-fold) serum-starved MSCs. The results were normalized to the positive controls. Similar results were obtained from three independent MSCs-CM samples. (B). TSG-6 in 30-folded serum-starved MSCs-CM within 24 h were measured by Enzyme-linked immunosorbent assays (ELISA). The concentration of TSG-6 in 12 h MSCs-CM was 0.75±0.09 ng/ml (49-fold increase, compared with 0 h MSCs-CM), 24 h MSCs-CM was 3.08±0.22 ng/ml (199.8-fold increase, compared with 0 h MSCs-CM). Three independent samples were placed in three repetitive holes. * compared with 0h MSCs-CM, *p* <0.05; # compared with 12 h MSCs-CM, *p* <0.05.

### Macroscopic evaluation of peritoneal adhesions

Rats were sacrificed at days 8, 10 or 14 after scraping (n  = 6 in each group of each time point). The size and severity of peritoneal adhesions were evaluated macroscopically by an independent observer on a scale of 0–4 (0, 0%; 1, <25%; 2, 25–49%; 3, 50–74%; and 4, 75–100% adhesions) using a previously reported scoring system [28].

**Figure 8:**
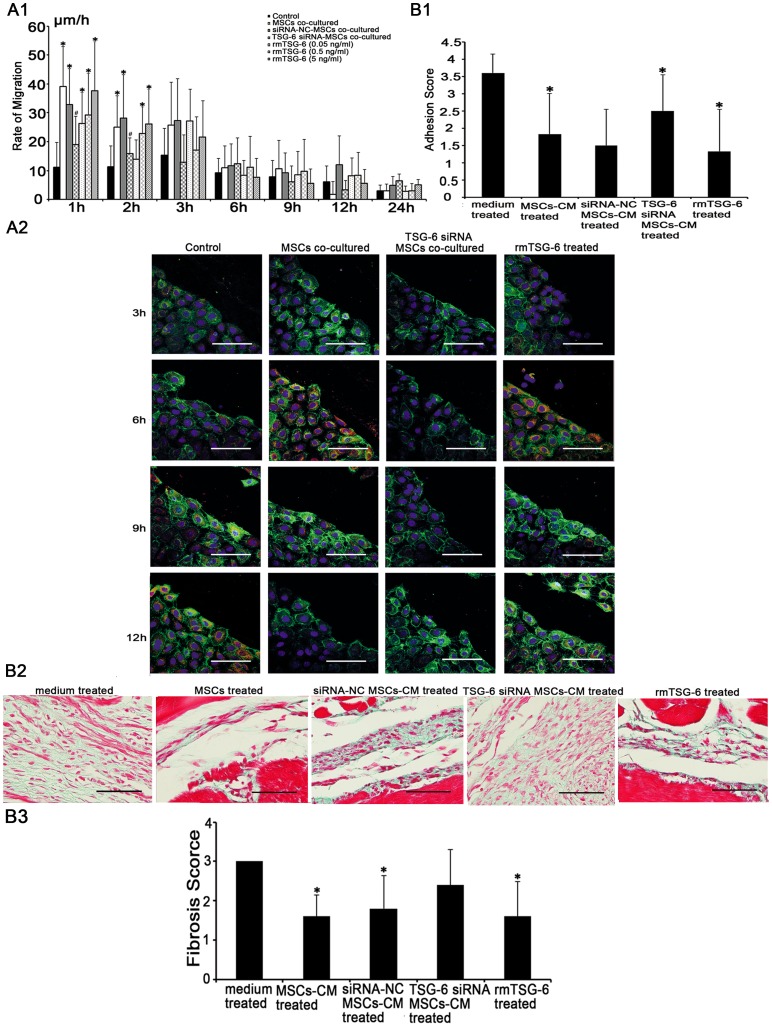
TNF-stimulating gene (TSG)-6 secreted by mesenchymal stem cells (MSCs) ameliorated the peritoneal injury. (A). The effects of TSG-6 on the repair of mechanically injured rat peritoneal mesothelial cells (RPMCs). (A1). Microscopy of RPMCs migrating to the edge of the wound to show the migratory velocity (μm/h). Injured RPMCs co-cultured with TSG-6-siRNA MSCs showed significant reductions in the migratory capacity. However, migrations were significantly increased dose-dependently in RPMCs cultured in medium containing recombinant mouse (rm) TSG-6. * compared with control group, *p* <0.05; # compared with MSCs co-cultured group, *p* <0.05; n  = 3, respectively. (A2). Immunofluorescence analysis with PCNA (red) and E-Cadherin (green) revealed the proliferation of injured RPMCs. RPMCs co-cultured with TSG-6-siRNA MSCs showed no apparent increase in the proliferative capacity. whereas the proliferation peak of RPMCs cultured with rmTSG-6 was accelerated to 6 h after scraping. Nuclei were stained with DAPI (blue). Magnification  = ×600. (B). The effects of TSG-6 on acute peritoneal adhesions. (B1). TSG-6-siRNA MSCs-CM treated group had no significant reduction in adhesion scores days 14 after scraping. However, adhesion scores were reduced in rmTSG-6 treated group. The size and severity of peritoneal adhesions were evaluated macroscopically by an independent observer on a scale of 0–4 (0, 0%; 1, <25%; 2, 25–49%; 3, 50–74%; and 4, 75–100% adhesions). * compared with medium treated group, *p* <0.05, n  = 6, respectively. (B2). Histological changes were evaluated using masson's trichrome staining. TSG-6-siRNA MSCs-CM treated group revealed no apparent reduction in fibrosis in the scraped peritoneum days 14 after scraping. However, fibrosis were reduced in rmTSG-6 treated group. Magnification  = ×400. (B3). The scores of peritoneal fibrosis days 14 after scraping were reduced by injecting rmTSG-6, but not by injecting TSG-6-siRNA MSCs-CM. * compared with medium treated group, *p* <0.05, n  = 6, respectively. The sections were evaluated from five randomly selected fields under a magnification of ×100 by an independent pathologist. The extent of fibrosis was scored as 0 (negative), 1 (weak), 2 (medium), or 3 (intensive).

### Histological analysis of peritoneal adhesions

Rats were sacrificed at days 2, 4, 6 or 14 after scraping (n  = 6 in each group of each time point). When fibrous bands did not form, parietal peritoneum was sampled, otherwise, the entire fibrous band was sampled. Specimens were fixed in 10% formaldehyde for 24 h. After dehydration, they were embedded in paraffin, and 3-μm thick cross-sections were stained with hematoxylin and eosin (HE) and/or masson's trichrome. Each tissue section was observed by light microscopy (Olympus IX71, Tokyo, Japan) at a magnification of ×100 and ×400. The sections were evaluated from five randomly selected fields by an independent pathologist (magnification of ×100). The extents of fibrosis (masson's trichrome staining) and inflammation (HE staining) were scored as 0 (negative), 1 (weak), 2 (medium), or 3 (intensive), respectively.

**Table 1:** Antibody based protein array of cytokine profile in serum-starved mesenchymal stem cells (MSCs)-conditioned medium (CM) within 24 h.

cytokines	0h	12h	24h	24h increase in fold
TSG-6	0.0004	0.0208	0.0766	194.0
LIX	0.0108	0.1869	0.5308	49.1
Fractalkine	0.0412	1.1578	1.7780	43.2
MMP-8	0.0004	0.0029	0.0157	41.1
CINC-3	0.0099	0.2074	0.3622	36.4
Fas Ligand	0.0022	0.0057	0.0719	32.5
MCP-1	0.0100	0.2950	0.3062	30.6
TIMP-1	0.0192	0.3700	0.5631	29.4
VEGF	0.0104	0.1680	0.2346	22.7
ICAM-1	0.0052	0.0511	0.0979	18.7
Activin A	0.0053	0.0097	0.0840	15.8
CINC-2 α	0.0419	0.7496	0.5287	12.6
PDGF-AA	0.0115	0.0928	0.1149	10.0
CINC-1	0.0326	0.2264	0.1939	5.9
MIP-3 α	0.0101	0.0159	0.0448	4.4
IL-10	0.0068	0.0096	0.0269	4.0
B7-2/CD86	0.0043	0.0061	0.0161	3.7
β-NGF	0.0203	0.0290	0.0752	3.7
GM-CSF	0.0079	0.0152	0.0259	3.3
IFN-γ	0.0006	0.0018	0.0019	3.1
RAGE	0.0036	0.0056	0.0096	2.7
IL-1 R6	0.0178	0.0225	0.0463	2.6
L-Selectin	0.0048	0.0057	0.0119	2.5
Thymus Chemokine-1	0.0179	0.0192	0.0445	2.5
IL-1 β	0.0067	0.0106	0.0153	2.3
IL-4	0.0113	0.0171	0.0246	2.2
IL-2	0.0139	0.0188	0.0286	2.0
TNF-α	0.0173	0.0279	0.0353	2.0
IL-6	0.0074	0.0088	0.0151	2.0
IL-1 α	0.0042	0.0053	0.0084	2.0
Leptin	0.0102	0.0126	0.0201	2.0
IL-13	0.0286	0.0348	0.0429	1.5
ADM	0.0104	0.0129	0.0155	1.5
HGF	0.0104	0.0129	0.0155	1.5
IGF-1	0.0104	0.0129	0.0155	1.5
Agrin	0.0078	0.0070	0.0110	1.4
Prolactin R	0.0097	0.0104	0.0129	1.3
CNTF	0.0086	0.0111	0.0096	1.1

The results were normalized to the positive controls. Similar results were obtained from three independent MSCs-CM samples. TSG-6, TNF-stimulating gene-6; LIX, lipopolysaccharide-induced CXC chemokine; MMP-8, matrix metalloproteinase-8; CINC, cytokine induced neutrophil chemoattractant; TIMP, tissue inhibitor of metalloproteinase; vascular VEGF, endothelial growth factor; ICAM, inter-cellular adhesion molecule; PDGF, platelet-derived growth factor.

### Immunohistochemical evaluation of peritoneal adhesions

Rats were sacrificed at days 2, 4, 6, or 14 after scraping (n  = 6 in each group of each time point). When fibrous bands did not form, parietal peritoneum was sampled, otherwise, the entire fibrous band was sampled. Briefly, 4-μm thick paraffin-embedded tissue sections mounted on poly-L-lysine precoated slides (ZSGB-Bio, Beijing, China) were deparaffinized in xylene and rehydrated in a graded alcohol series. The slides were treated with 3% hydrogen peroxide for 10 min at 4°C and washed with PBS for 5 min. After microwave antigen retrieval, the slide was blocked with 1% bovine serum albumin (BSA) for 30 min at room temperature. The following primary antibodies were incubated overnight at 4°C: E-Cadherin (1∶100) (Abcam, Cambridge, UK), MPO (1∶50) (Santa Cruz Biotechnology, Santa Cruz, CA), ED-1 (1∶50) (Santa Cruz Biotechnology, Santa Cruz, CA), FSP-1 (1∶50) (Thermo Fisher Scientific, Fremont, CA) and TGF-β1 (1∶50) (Santa Cruz Biotechnology, Santa Cruz, CA). After washing with PBS, HRP-conjugated secondary antibodies (Dako, Glostrup, Denmark) were applied for 1 h at room temperature. The sections were then washed with PBS and incubated with avidin-biotin peroxidase conjugate (ABC Kit, Vector Laboratories, Burlingame, CA) at a dilution of 1∶100 for 30 min at room temperature, after which ABC substrate (ABC Kit, Vector Laboratories, Burlingame, CA) was added for 5–10 min. Nuclei were counterstained with Carazzi hematoxylin. Negative controls did not receive the first antibody. Each tissue section was observed by light microscopy at a magnification of ×100 and ×400. Sections were evaluated from five randomly selected fields by an independent pathologist (magnification of ×100). The extent of staining was scored as 0 (0%), 1 (1–20%), 2 (21–50%), 3 (51–80%), and 4 (81–100%), indicating the percentage of positive staining in the adhesion tissue.

### Immunofluorescence staining of peritoneal adhesions

Rats were sacrificed at days 2, 4, or 6 after scraping (n  = 6 in each group). When fibrous bands did not form, parietal peritoneum was sampled, otherwise, the entire fibrous band was sampled. Specimens were fixed in 4% paraformaldehyde at 4°C for 4 h followed by graded dehydration in 30, 20, and 10% sucrose for 30 min. The specimens were then embedded in O.C.T. compound (Sakura Finetek USA, Torrance, CA) and stored at −80°C until use. Frozen tissues were sectioned every 4 μm and placed on poly-L-lysine precoated slides. The slides were washed three times with PBS and then blocked with 1% BSA for 30 min at room temperature. The following primary antibodies were incubated overnight at 4°C: E-Cadherin (1∶100) and PCNA (1∶100) (Cell Signaling Technology, Danvers, MA). After washing with PBS, secondary antibodies conjugated with FITC or Cy3 (Jackson ImmunoResearch Laboratories, West Grove, PA) were applied for 1 h at room temperature in a darkened humidified chamber. Finally, the preparations were washed with PBS and mounted in fluorescent mounting medium with DAPI (ZSGB-Bio, Beijing, China). Negative controls did not receive the first antibody. Each tissue section was observed under a confocal laser scanning microscope (Olympus FluoView 1000, Tokyo, Japan) at a magnification of ×600 and ×1800, if necessary.

### Isolation and culture of RPMCs

RPMCs were isolated and cultured according to a previous method [29]. Briefly, RPMCs were obtained by infusing 30 ml of 0.25% trypsinase-0.2% EDTA-Na_2_ into the rat abdominal cavity. The fluid was collected from the peritoneal cavity 1 h later under sterile conditions. Cellular components were isolated by centrifugation at 1400 rpm for 8 min and then washed with PBS and suspended in DMEM/F12 medium (Gibco BRL, Grand Island, NY) supplemented with 12% (v/v) fetal calf serum (Gibco BRL, Grand Island, NY). The cells were placed in 25-cm^2^ culture flasks and incubated overnight at 37°C under 5% CO_2_ and 90% humidity. Nonadherent cells were removed two days later with two brief PBS washes, and the adherent cells were incubated in fresh DMEM/F12 medium. The cells reached confluence in five days. The RPMCs in this study were derived from fourth-eighth passages grown as a monolayer to subconfluency.

### Immunofluorescence staining of RPMCs

Cells were fixed with freshly prepared 4% paraformaldehyde for 10 min at room temperature. The cells were then washed three times with PBS. Each cover slip was then incubated in 1% BSA. The following primary antibodies were incubated overnight at 4°C: E-Cadherin (1∶100), basic Cytokeratin (1∶50) (Santa Cruz Biotechnology, Santa Cruz, CA), α-SMA (1∶100) (Sigma-Aldrich Corp, St. Louis, MO) and PCNA (1∶100). After washing with PBS, secondary antibodies conjugated with FITC or Cy3 were applied for 1 h at room temperature in a darkened humidified chamber. Finally, the preparations were washed with PBS and mounted in fluorescent mounting medium with DAPI. Negative controls did not receive the first antibody. Each tissue section was observed under a confocal laser scanning microscope at a magnification of ×200 and ×600, if necessary.

### RPMCs scraping model

RPMCs were scraped based on a previous method [25] and grown to confluence on glass coverslips placed in a twelve-well plate (1.5×10^5^/well) (Corning, Lowell, MA). Growth was arrested by incubation for 48 h in serum-free DMEM/F12 medium. Under this condition cells remained viable in a nonproliferating state. The quiescent monolayer was injured by scraping with a sterile glass rod (φ  = 1 mm). The monolayer was washed twice with PBS and then incubated with serum-free DMEM/F12 medium, rmTSG-6 (0.05 (corresponding to the level of TSG-6 produced by 5×10^4^ MSCs), 0.5, 5 ng/ml, respectively) [30] or co-cultured with MSCs, TSG-6-siRNA MSCs (5×10^4^) seeded on transwell inserts (polyester membrane, pore size  = 0.4 μm) (Corning, Lowell, MA). Closure of the denuded areas was monitored by light microscopy at a magnification of ×100. At 1, 2, 3, 6, 9, 12, 24, 36, and 48 h after scraping, images of the wounded areas were captured, and the motility rates of cells entering the wound (μm/h) were calculated from ten separate points along the original margin of the wounds. The cells were stained as described above for immunofluorescence staining.

### In vivo imaging and immunofluorescence evaluation of MSCs after injection

MSCs was labeled with near-infrared fluorescence lipophilic DiR (Molecular Probes, Eugene, OR) before intravenous or intraperitoneal injection. Briefly, MSCs were trypsinized and resuspended at a density of 1×10^6^/ml in serum-free DMEM containing 5 μg of DiR. The mixed solution was incubated for 30 min at 37°C and 4°C for 15 min. The cells were then washed three times with PBS and the viability of labeled cells was verified by trypan blue staining. IVIS^®^ Lumina II *in vivo* imaging system (Caliper Life Sciences, Hopkinton, MA) was used to follow the redistribution of MSCs over time *in vivo*. The excitation and emission filter set in the IVIS was 745 nm and 780 nm, respectively. MSCs (5×10^6^) were injected into rats via tail vein or peritoneum at 24 h after scraping and assayed for up to seven days thereafter (0, 0.5, 1, 2, 4, 8, 12, 24, 72 h and 7 days) until the fluorescence became undetectable. GFP immunofluorescence was used to detect the distribution of MSCs in specific tissues. Rats were sacrificed at 0, 4, 6, 8, 12 and 24 h after MSCs injection (n  = 3 in each group of each time point). The right lower lung was sampled, and the specimens were embedded in O.C.T. compound and stored at −80°C until use. Frozen tissues were sectioned every 4 μm. The slides were washed three times with PBS and then blocked with 1% BSA for 30 min at room temperature. The GFP primary antibodies (1∶100) (Santa Cruz Biotechnology, Santa Cruz, CA) were incubated overnight at 4°C. After washing with PBS, secondary antibodies conjugated with Cy3 were applied for 1 h at room temperature in a darkened humidified chamber. Finally, the preparations were washed thoroughly with PBS and mounted in fluorescent mounting medium with DAPI. Each tissue section was observed with a confocal laser scanning microscope at a magnification of ×1000.

### Cytokine Array of MSCs-CM

Antibody based cytokine array analysis of 30-fold concentrated MSCs-CM (0h, 12 h, 24 h serum-starved respectively) was performed using a RayBio^®^ Rat Cytokine Antibody Array G Series (detecting 34 proteins) and a RayBio^®^ Label-based Rat Antibody Array (detecting hepatic growth factor (HGF), insulin-like growth factor (IGF)-1, adrenomedulin (ADM) and TSG-6) (Raybiotech Inc., Norcross, GA). The analysis was performed using the manufacturer's recommended protocol, and the signals of Cy3 were imaged by Axon GenePix laser scanner (MDS analytical technologies, Sunnyvale, CA).

### ELISA of cytokines in CM

Quantification of TSG-6 in CM (0 h, 12 h, 24 h serum-starved or TSG-6-siRNA, siRNA-NC MSCs produced) was performed by ELISA according to the manufacturer's instructions. Absorbance was measured at 450 nm using a microplate reader (MULTISKAN MK3, Thermo Fisher Scientific, Waltham, MA) . TSG-6 concentrations were determined with a standard curve constructed by titrating standard TSG-6.

### Statistical Analysis

Analysis was done using IBM^®^ SPSS^®^ Statistics 17.0.2 software (IBM Corporation, Armonk, NY). Results were presented as mean values±standard deviation. Multiple comparisons of parametric data were performed using one-way analysis of variance (ANOVA), followed by Student-Newman-Keuls post-hoc tests. Nonparametric data were compared with the Mann-Whitney *U*-test to identify differences between groups, and α was corrected by the numbers of comparisons (α/comparisons) to ensure α  = 0.05. *p* <0.05 was considered statistically significant for all analysis.

## Results

### MSCs injected via tail vein ameliorated the inflammation and fibrosis of acute peritoneal adhesions

On days 8, 10 and 14 after peritoneal scraping, the rats administered serum-free medium demonstrated dense adhesions, with higher mean adhesion scores. The rats treated with MSCs injected via tail vein showed reductions in adhesions. We observed statistically significant differences among the groups for the size and severity of adhesions (Figure 1A) (mean score: 8 days, 3.4 *vs*. 1.4; 10 days, 3.6 *vs*. 1.8; 14 days, 3.6 *vs*. 2.0; *p* <0.05, n  = 6, respectively). Histological HE staining (Figure 1B1 and Figure S1A) and immunohistochemistry (Figure 2A and Figure S2) revealed exudative changes and infiltration of neutrophil inflammatory cells into the mesothelial and interstitial spaces 2 days after scraping,with an increase in the number of macrophage and the level of TGF-β1 4 days after scraping. Histological masson's trichrome staining (Figure 1C1 and Figure S1B) showed that the inflammatory process led to nascent fibrinous adhesions. Over 14 days, the number of fibroblasts decreased and the adhesions matured into fibrous bands containing scarce cells, adipose tissue, blood vessels, and collagen. MSCs injection decreased the inflammations in active phase (Figure 1B1 and Figure 1B2) (mean score: 2 days, 2.4 *vs*. 1.3; *p* <0.05, n  = 6, respectively). For example, MSCs reduced the infiltration of neutrophils (Figure 2A and Figure 2B) (mean score: 2 days, 4.0 *vs*. 2.8; 4 days, 3.6 *vs*. 2.2; *p* <0.05, n  = 6, respectively), and macrophages (mean score: 6 days, 2.8 *vs*. 1.2; *p* <0.05, n  = 6, respectively). In addition, MSCs also reduced the number of fibroblasts (Figure 2A and Figure 2B) (mean score: 4 days, 3.4 *vs*. 1.8; 6 days, 3.6 *vs*. 1.8; *p* <0.05, n  = 6, respectively), and the level of TGF-β1 (mean score: 4 days, 2.4 *vs*. 1.4; 6 days, 3.8 *vs*. 1.8; *p* <0.05, n  = 6, respectively). Consequently, the deposition of collagen was also decreased (Figure 1C1 and Figure 1C2) (mean score: 4 days, 1.7 *vs*. 1.3; 6 days, 2.8 *vs*. 1.8; 14 days, 2.8 *vs*. 1.6; *p* <0.05, n  = 6, respectively).

### MSCs accelerated the repair of peritoneal mesothelial cells

Immunohistochemical (Figure 3A1 and Figure S2) and immunofluorescence evaluations (Figure 3B) revealed the proliferation and repair of mesothelial cells. MSCs seemed to accelerate mesothelial cell repair (Figure 3A1 and Figure 3A2) (mean score: 2 days, 1.2 *vs*. 2.4; 4 days, 1.4 *vs*. 2.6; *p* <0.05, n  = 6, respectively). Dual-stained E-Cadherin- and PCNA-positive mesothelial cells in the mesothelial and submesothelial layers were more prominent in MSCs treated group 4 days after peritoneal scraping, compared with medium treated group.

### MSCs accelerated the repair of mechanically injured RPMCs in vitro

Primary RPMCs grew to confluence over five days when a non-overlapping monolayer of cobblestone-like epithelial cells appeared (Figure 4A1). Immunofluorescence analysis revealed that the cells were mesothelial cells based on positive staining for E-Cadherin and Cytokeratin and weak staining for α-SMA (Figure 4A2). Mechanical injury of the cultured cells served as a useful *in vitro* system for studying the response of cells to injury [25], [31]. The mean widths of a series of wounds measured from light photomicrographs at time zero were 0.243±0.043 mm in control group (n  = 3) and 0.240±0.029 mm in the co-culture group (n  = 3). During the first 2 h after scraping, a distinct increase in the migration of the RPMCs was observed when the cells were incubated in the transwell with MSCs compared with control group (1 h, 21.763±2.807 μm/h *vs*. 11.057±5.456 μm/h; 2 h, 21.993±2.720 μm/h *vs*. 10.483±6.606 μm/h; *p* <0.05, n  = 3, respectively) (Figure 4B1). Immunofluorescence analysis of PCNA demonstrated that the proliferation of injured RPMCs co-cultured with MSCs increased dramatically 3 h after scraping; peak proliferation occurred 6 h after scraping. In control group, proliferation increased 9 h after scraping, with a peak at 12 h after scraping (Figure 4B2).

### MSCs did not accumulate in the injured peritoneum

An *in vivo* imaging system was used to follow the distribution of MSCs labeled with DiR after injection into rat tail vein. MSCs first accumulated in the lungs 1 h after injection; the fluorescence signal reached its maximum intensity at 4 h and persisted for at least seven days. At 6 h after injection, the signal began to accumulate in the liver and the spleen (Figure 5A1), no signal was found in the injured peritoneum throughout the entire period. When MSCs were injected intraperitoneally, MSCs first accumulated in the liver 1 h after injection and gradually in the spleen, which persisted for at least seven days (Figure 5A2), but no apparent signal was found in the injured peritoneum. Immunofluorescence analysis did not detect significant engraftment of MSCs/GFP in the injured peritoneum. Moreover, lung histological sections demonstrated that MSCs trapped in the lungs during the first 24 h (Figure 5B). Allogeneic MSCs are reported not to be immune-privileged, but they may elicit a memory response leading to rapid clearance of subsequent doses by the immune system [32]. Therefore, we injected MSCs intravenously or intraperitoneally into SCID mice 24 h after scraping and found similar negative results in the injured peritoneum (data not shown).

### MSCs-CM injected via tail vein reduced the formation of acute peritoneal adhesions

We serum-starved MSCs as a pretreatment to maximize their protective properties. To ensure a persistent effect, rats were treated with MSCs-CM via tail vein injection three times within seven days after scraping. These rats showed a reduction in adhesion on days 8, 10, and 14 after peritoneal scraping (Figure 6A). We observed statistically significant differences in the size and severity of adhesions, compared with those in medium treated group (mean score: 8 days, 1.6 *vs*. 3.4; 10 days, 2.0 *vs*. 3.6; 14 days, 2.1 *vs*. 3.6; *p* <0.05, n  = 6, respectively), but no differences were observed between MSCs treated group and MSCs-CM treated group (mean score: 8 days, 1.4 *vs*. 1.6; 10 days, 1.8 *vs*. 2.0; 14 days, 2.0 *vs*. 2.1; *p* >0.05, n  = 6, respectively). Histological masson's trichrome staining revealed that MSCs-CM reduced collagen deposition (Figure 6B1, Figure 6B2 and Figure S3) (mean score: 8 days, 2.6 *vs*. 1.4; 14 days, 2.8 *vs*. 1.6; *p* <0.05, n  = 6, respectively), similar to MSCs (Figure 6B1, Figure 6B2 and Figure S3) (mean score: 8 days, 1.4 *vs*. 1.5; 14 days, 1.6 *vs*. 1.6; *p* > 0.05, n  = 6, respectively).

### Cytokine profile in serum-starved MSCs-CM

To examine protein levels of cytokine released by MSCs, we performed antibody based protein array analysis of MSCs-CM under serum-starving condition within 24 h. Compared with 0 h MSCs-CM, 13 cytokines were apparently higher in MSCs-CM (≥5-fold, compared with 0 h), which were released abundantly within either 12 h or 24 h, including TSG-6, lipopolysaccharide-induced CXC chemokine (LIX), Fractalkine, matrix metalloproteinase (MMP)-8, cytokine induced neutrophil chemoattractant (CINC)-3, Fas Ligand, tissue inhibitor of metalloproteinase (TIMP)-1, vascular endothelial growth factor (VEGF), inter-cellular adhesion molecule (ICAM)-1, Activin A, CINC-2α, platelet-derived growth factor (PDGF)-AA, CINC-1 (Figure 7A and Table. 1). 24 h MSCs-CM had abundant levels of TSG-6 (194-fold compared with 0 h), which is known to be important in wound healing [21], [22]. This cytokine was further measured with ELISA. The result revealed that 24 h MSCs-CM had 199.8-fold increase in TSG-6 concentration (3.08±0.22 ng/ml) , compared with 0 h (Figure 7B), which was similar to that of protein array analysis.

### TSG-6 of MSCs was involved in the reduction of peritoneal injury

To further evaluate the role of TSG-6 secreted by MSCs in the reduction of perotoneal injury, we knocked down the expression of TSG-6 in MSCs by transient transfection with a TSG-6-siRNA (Figure S4). *In vitro*, compared with MSCs co-cultured group, there were significant reductions in the migratory capacity of the scraped RPMCs co-cultured with TSG-6-siRNA MSCs during the first 2 h after scraping (Figure 8A1) (1 h, 39.073±13.806 μm/h *vs.* 19.100±9.692 μm/h; 2 h, 25.056±10.885 μm/h *vs*. 15.997±5.363 μm/h; *p* <0.05, n  = 3, respectively). However, compared with control group, the migration of RPMCs cultured with rmTSG-6 (0.05, 0.5, 5 ng/ml, respectively) were significantly increased dose-dependently within 2 h after scraping (Figure 8A1) (1 h, 11.148±8.550 μm/h *vs.* 26.548±10.542 μm/h, 29.467±14.282 μm/h, 37.829±16.765 μm/h, respectively; 2 h, 11.265±7.325 μm/h *vs.* 14.177±6.344 μm/h, 23.050±9.033 μm/h, 26.291±11.875 μm/h, respectively; *p* <0.05, n  = 3, respectively). The proliferation of RPMCs cultured with rmTSG-6 (5 ng/ml) increased dramatically 3 h after scraping with a peak at 6 h, which was similar to MSCs co-cultured group; while the proliferation of RPMCs co-cultured with TSG-6-siRNA MSCs was not accelerated (Figure 8A2). *In vivo*, compared with medium treated group, TSG-6-siRNA MSCs-CM had no significant effect on the size, severity (Figure 8B1) (mean score: 3.6 *vs*. 2.5, *p* >0.05, n  = 6, respectively) or the histological changes (Figure 8B2, Figure 8B3 and Figure S5) (mean score: 3.0 *vs*. 2.4, *p* > 0.05, n  = 6, respectively) of peritoneal adhesions 14 days after scraping. However, compared with medium treated group, both the size, severity of peritoneal adhesions (Figure 8B1) (mean score: 3.6 *vs*. 1.3, *p* <0.05, n  = 6, respectively) and the collagen depositions were reduced in rats injected with medium containing rmTSG-6 (3 ng/ml) (Figure 8B2, Figure 8B3 and Figure S5) (mean score: 3.0 *vs*. 1.6, *p* <0.05, n  = 6, respectively).

## Discussion

To investigate the effects of MSCs on injured peritoneal mesothelial cells and peritoneal fibrosis, we established a well-documented acute peritoneal adhesion rat model [24]. Scraping caused injury to the mesothelial cell layer, leading to neutrophil infiltration during the first 12 h (data not shown), followed by macrophage recruitment and the release of fibrinous exudates into the peritoneum. This process led to the formation of nascent fibrinous adhesions 4 days after scraping. Seven days later, the number of fibroblasts decreased and the adhesions matured into fibrous bands with scarce cells. TGF-β1 is involved in wound-healing processes and favors the formation of peritoneal fibrosis and adhesions [3], [33]. TGF-β1 increased to a peak six days after scraping and decreased gradually thereafter. Mesothelial cell deposition at the submesothelial zone commenced 24 h after scraping and finished within seven days, similar to previous report [34].

Recent studies have demonstrated that MSCs participate in tissue repair [9]. Moreover, MSCs decrease tissue fibrosis [10]–[13]. We tested commercial MSCs/GFP (tenth passage) by FACS to examine the expression of MSCs surface markers. The cells displayed high levels of CD54 and CD90 and an absence of CD11a or CD45. Upon the application of adipogenic and osteogenic differentiation conditions, the cells accumulated intracellular lipid droplets, as revealed by Oil red staining, and displayed extracellular calcium phosphate precipitates as identified by von Kossa staining (Figure S6).

According to our previous experiments, we determined the optimal therapeutic strategy, that is, 5×10^6^ MSCs were injected via tail vein 24 h after scraping. Because the pathological changes stabilized seven days after scraping, we evaluated the peritoneal adhesions at days 8, 10, and 14 after scraping. MSCs reduced the infiltration of neutrophils and macrophage cells, the number of fibroblasts, the level of TGF-β1, and increased the proliferation of mesothelial cells, consequently, ameliorated fibrinous deposition in adhesive bands. We also established a mechanical injury model of cultured cells *in vitro*. Because the basic experimental conditions employed homogenous RPMCs and was undertaken under conditions without serum, the model provided information on the direct response of RPMCs to injury [25]. We found that MSCs could increase the early (the first 2 h) migratory capacity of RPMCs and accelerate the proliferation 6 h ahead of control group. A recent study showed that peritoneal adhesions were attenuated by enhancing the proliferation and migration of mesothelial cells [6]. But our study avoided any potential contribution from the other cells located in the peritoneum, such as macrophages or fibroblasts. Besides, extensive evidence show that a comprehensive cytokine network play a critical role in the repair of mesothelial cells [35]. Therefore, *in vivo*, the potential contributions from other cells and cytokines should be considered. This might explain for the phenomenon that the proliferative effect of MSCs on RPMCs *in vitro* did not correlate with the data obtained *in vivo*.

The mechanisms by which MSCs exert their beneficial effects remain controversial. Studies have postulated that MSCs mediate their therapeutic effects by either differentiating into functional reparative cells that replace injured tissues or by secreting paracrine factors that promote repair [18], [36]. MSCs injected intraperitoneally did not ameliorate peritoneal adhesions (Figure S7). Then we tracked the dynamic distribution of MSCs after their injection into rats via tail vein or peritoneum. MSCs accumulated in the lungs first and gradually accumulated in the liver and spleen; however, no apparent cells were observed in the injured peritoneum even when MSCs were injected intraperitoneally. Recent studies have found that the vast majority of MSCs injected intravenously home to the vascular endothelium of the lungs and liver [37], [38], where they appear as emboli in afferent blood vessels [37], [39]. This distribution may be due to the size of MSCs (20–30 μm) relative to pulmonary capillaries (14 μm in diameter), which may prevent the infused MSCs from passing through the pulmonary circulation. We speculated that MSCs injected into the peritoneal cavity might be absorbed through veins or lymphatic tubes and accumulated in the liver and spleen. Phagocytic response in the monocyte-macrophage system might do damage to MSCs. It is worth mentioning that allogeneic MSCs are not immunoprivileged; they may elicit a memory response leading to rapid clearance by the immune system [32]. We injected MSCs intravenously or intraperitoneally into SCID mice 24 h after peritoneal scraping and found similar negative results in the injured peritoneum (data not shown). Therefore, the acquired immune system may not influence the fate of MSCs in our rat model. One possible explanation is that ROS inhibited the cellular adhesion of engrafted MSCs [40]. Further investigations must be performed to explain this interesting phenomenon.

It has become apparent that MSCs repair injured tissues without significant engraftment or differentiation in some situations [37], [41]. In fact, MSCs secrete a number of cytokines and growth factors that alter the tissue microenvironment, such as TSG-6 [22], [37], VEGF _ENREF_38[16] and PDGF [17]. One possibility is that the cells trapped in the lungs secrete soluble factors into the blood to enhance the repair of other tissues by suppressing inflammatory and immune reactions or by stimulating the propagation and differentiation of tissue-endogenous stem cells [37], [42]. MSCs secrete a wide spectrum of biologically active factors that can be found in CM [43]. Some studies have suggested that pretreatment with serum-starved MSCs may maximize their protective properties [27], [44]. We injected serum-starved MSCs-CM into rats via tail vein and found that MSCs-CM reduced adhesion formation, similar to MSCs.

We explored the cytokine profile released by serum-starved MSCs within 24 h using antibody based protein array analysis. Besides some inflammatory and chemotactic factors, we also found wound healing cytokines VEGF, PDGF-AA, especially TSG-6 (194-fold compared with 0 h MSCs-CM, was further measured with ELISA) were released abundantly. TSG-6 is a 35 kDa hyaluronan (HA)-binding glycoprotein with multifunctional anti-inflammatory effects. In transgenic mice, inactivation of the gene increased inflammatory responses, and over-expression of the gene decreased inflammatory responses [22]. We knocked down the expression of TSG-6 in MSCs by a TSG-6 siRNA. TSG-6-siRNA MSCs failed to, but exogenous administration of TSG-6 could promote mesothelial repair and reduce peritoneal adhesions. Though some factors in addition to TSG-6 may be critical to the tissue repair, our data demonstrated that TSG-6 secretion by MSCs was both necessary and sufficient to promote the repair of peritoneal injury.

MSCs block the recruitment of neutrophils by secreting TSG-6 [22], most likely via a CD44/HA/TSG-6 mediated blocking mechanism [45]. Research found that neutrophils of thioglycollate-induced peritonitis were higher in TSG-6-deficient animals than in wild-type animals, but was dramatically suppressed by intravenous injection of rmTSG-6 [46]. Macrophages remain in the fibrinous zone for a longer time and also contribute to fibrosis [47]. TSG-6 interacts through the CD44 receptor on macrophages to decrease zymosan/TLR2 mediated nuclear translocation of NF-κB [22]. Moreover, TSG-6 up-regulates cyclooxygenase (COX)-2 expression in macrophages accompanied by an increase in the production of PGD_2_ which acts as a negative regulator of macrophage activation and inﬂammatory cytokines production in monocytes [48]. *In vitro*, RPMCs respond to injury by secreting IL-1, IL-6 and synthesizing increased amounts of proteoglycans and HA [25]. MSCs may be activated by this initial inflammatory microenvironment to secrete TSG-6. HA to its principle receptor, CD44, activates the mitogen-activated protein kinase (MAPK) pathway [49] and enhances migration and proliferation in cells following injury [50]. TSG-6-mediated formation of heavy chain-HA complexes is in involved in remodeling ECM and regulating cell migration and proliferation [49], [51]. Therefore, we suggested that TSG-6 secreted by MSCs could protect the injured peritoneum from the excessive inﬂammatory response and promote the repair of mesothelial cells, subsequently, reduce the formation of fibrosis.

In summary, our data demonstrate that MSCs attenuate peritoneal injury by stimulating the repair of mesothelial cells, reducing the inflammation and fibrosis of injured peritoneum. Rather than the engraftment, the secretion of TSG-6 by MSCs makes a major contribution to the therapeutic benefits of MSCs.

## Supporting Information

Figure S1 **Effects of mesenchymal stem cells (MSCs) on the histological changes of acute peritoneal adhesions.** (A). HE staining revealed changes in peritoneal inflammation after scraping, which were reduced by injecting MSCs. Magnification  = ×100. (B). Masson's trichrome staining revealed changes in peritoneal fibrosis after scraping, which were reduced by injecting MSCs. Magnification  = ×100.
(TIF)

Figure S2 **Evaluation of the effects of mesenchymal stem cells (MSCs) on the inflammation, fibrosis and peritoneal mesothelial cells of acute peritoneal adhesions.** Immunohistochemical evaluation revealed that the number of fibroblasts (FSP-1), neutrophils (MPO), and macrophage cells (ED-1), and the level of transforming growth factor (TGF)-β1 during the active phase were decreased by injecting MSCs, while the number of mesothelial cells (E-Cadherin) were increased by injecting MSCs. Magnification  = ×100.
(TIF)

Figure S3 **Evaluation of the effects of mesenchymal stem cells (MSCs)-conditioned medium (CM) on acute peritoneal adhesions.** Masson's trichrome staining revealed that the fibrosis in the scraped peritoneum was decreased by injecting MSCs-CM. Magnification  = ×100.
(TIF)

Figure S4 **The knockdown efficiency of TNFα-stimulating gene (TSG)-6 in mesenchymal stem cells (MSCs).** (A). Knockdown efficiency of mRNA level in MSCs was approximately 82.9% evaluated by reverse-transcriptase polymerase chain reaction (RT-PCR), TSG-6 product length  = 134 bp, GAPDH product length  = 87 bp. * compared with normal MSCs, *p* <0.05; # compared with 24 h sreum-starved MSCs, *p* <0.05. (B). Knockdown efficiency of protein level in MSCs-conditioned medium (CM) was approximately 73.4% evaluated by Enzyme-linked immunosorbent assay (ELISA). * compared with 0h MSCs-CM, *p* <0.05; # compared with 24 h MSCs-CM, *p* <0.05.
(TIF)

Figure S5 **Evaluation of the role of TNF-stimulating gene (TSG)-6 in the reduction of acute peritoneal adhesions by mesenchymal stem cells (MSCs).** Histological changes were evaluated using masson's trichrome staining. TSG-6-siRNA MSCs-CM treated group revealed no apparent reduction in the fibrosis of scraped peritoneum. However, the fibrosis was reduced in recombinant mouse (rm) TSG-6 treated group. Magnification  = ×100.
(TIF)

Figure S6 I**dentification of Sprague-Dawley (SD) rat bone marrow-derived mesenchymal stem cells (MSCs)/green fluorescent protein (GFP).** (A). Representative markers characteristic of MSCs. Fluorescence activated cell sorting (FACS) analysis was performed to examine the surface markers of MSCs. The positive proportion of cells displaying CD90 was 97.0%, CD54 was 88.8%, CD11a was 10.7% and CD45 was 8.4%. (B). Multilineage differentiation of MSCs. (B1). Osteogenic differentiation of MSCs. Under osteogenic differentiation conditions, the cells displayed extracellular calcium phosphate precipitates as identified by von Kossa staining. Magnification  = ×100. (B2). Adipogenic differentiation of MSCs. Under adipogenic differentiation conditions, the cells accumulated intracellular lipid droplets as revealed by Oil red staining. Magnification  = ×400.
(TIF)

Figure S7 **Evaluation of the effects of intraperitoneally injected mesenchymal stem cells (MSCs) on acute peritoneal adhesions.** Only MSCs injected intravenously group had lower adhesion scores. The size and severity of peritoneal adhesions were evaluated macroscopically by an independent observer on a scale of 0–4 (0, 0%; 1, <25%; 2, 25–49%; 3, 50–74%; and 4, 75–100% adhesions). * compared with medium treated group (iv), *p* <0.05, n  = 6, respectively.
(TIF)
