# Supplementary figures and images for: Mesenchymal Stem Cells Attenuate Peritoneal Injury through Secretion of TSG-6
Source: PLoS One. 2012 Aug 17;7(8):e43768. doi: 10.1371/journal.pone.0043768 (PMC3422344; doi:10.1371/journal.pone.0043768)

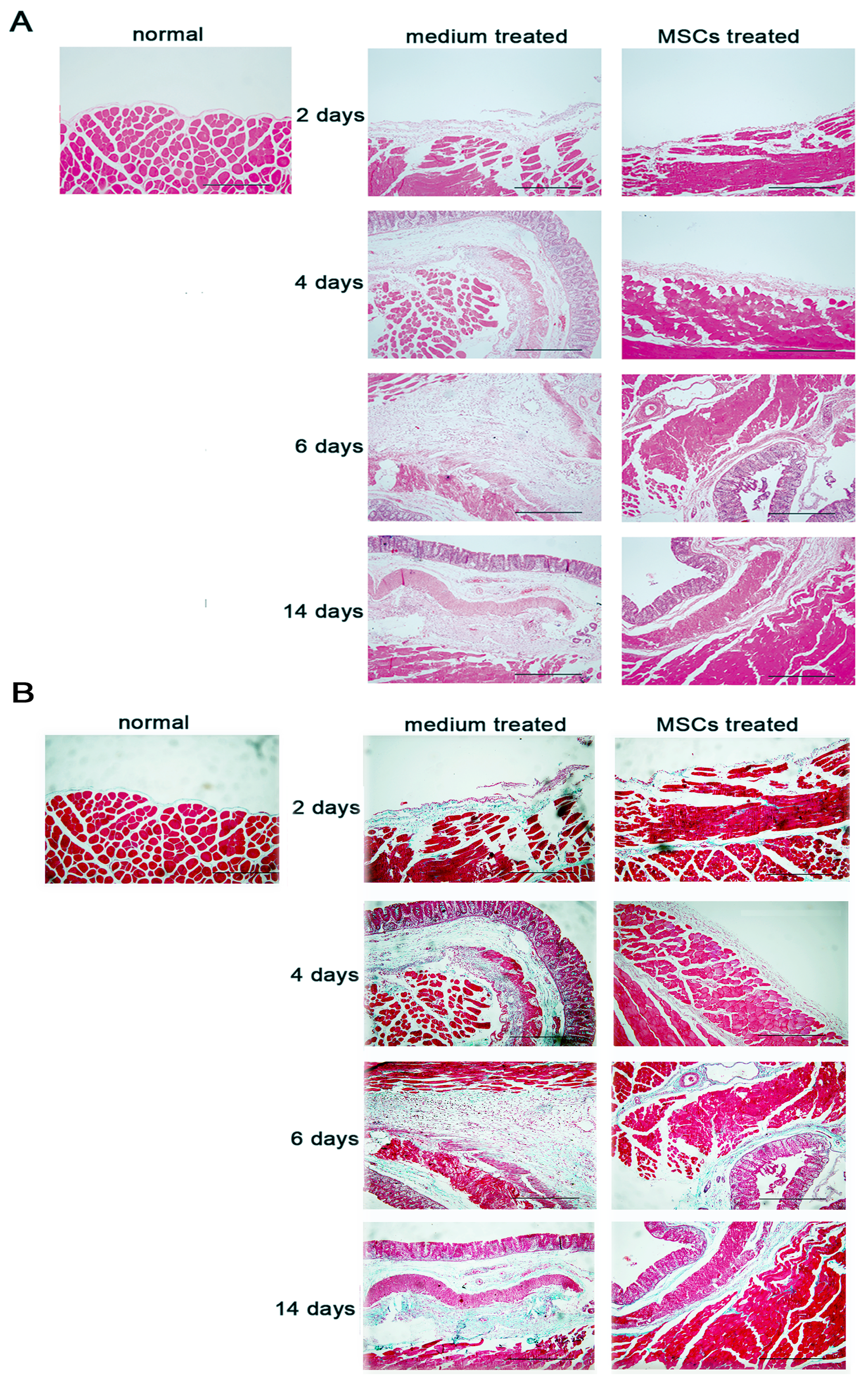

Supplement: Figure S1 — Effects of mesenchymal stem cells (MSCs) on the histological changes of acute peritoneal adhesions. (A). HE staining revealed changes in peritoneal inflammation after scraping, which were reduced by injecting MSCs. Magnification = ×100. (B). Masson's trichrome staining revealed changes in peritoneal fibrosis after scraping, which were reduced by injecting MSCs. Magnification = ×100. (TIF) [file pone.0043768.s001.tif]

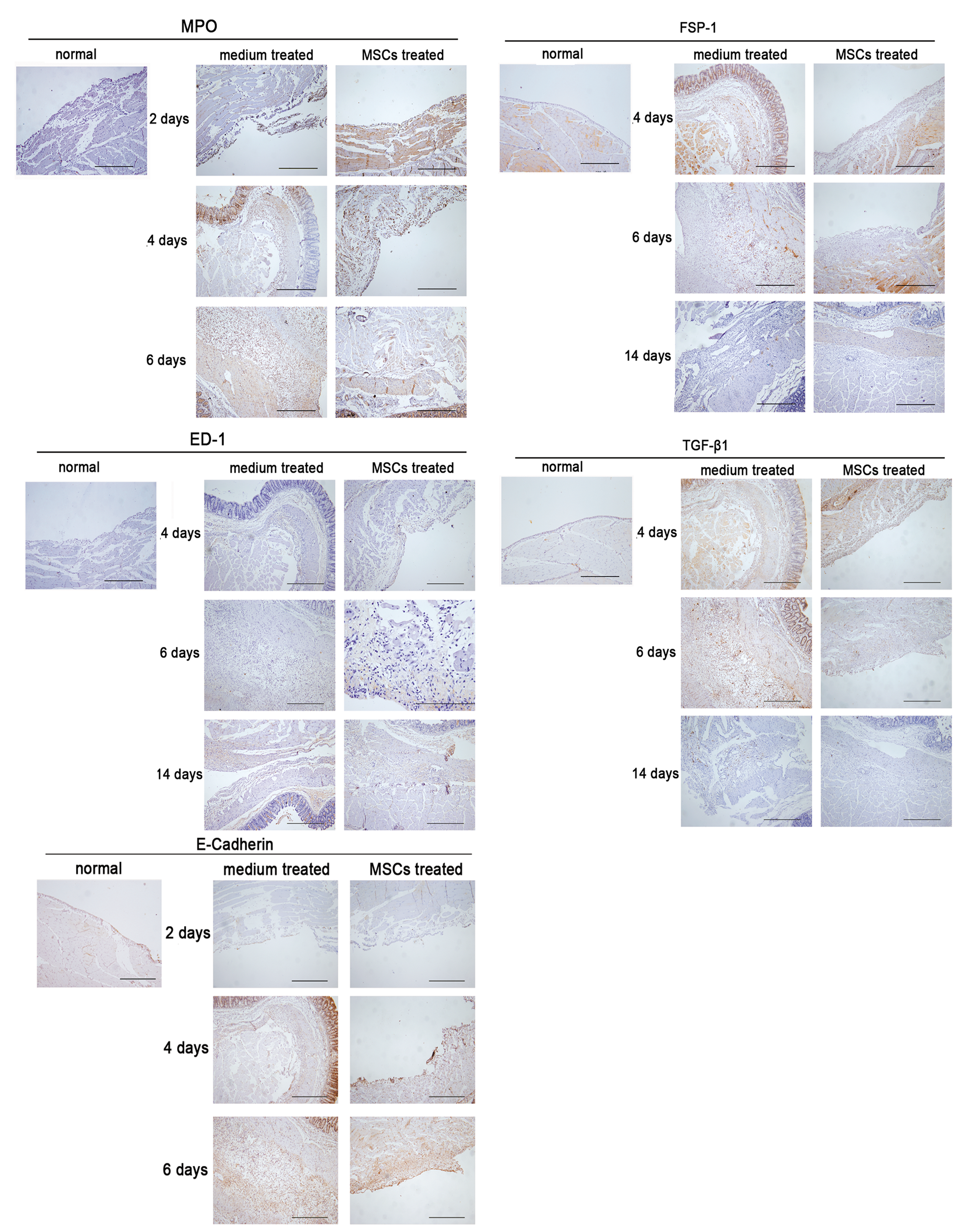

Supplement: Figure S2 — Evaluation of the effects of mesenchymal stem cells (MSCs) on the inflammation, fibrosis and peritoneal mesothelial cells of acute peritoneal adhesions. Immunohistochemical evaluation revealed that the number of fibroblasts (FSP-1), neutrophils (MPO), and macrophage cells (ED-1), and the level of transforming growth factor (TGF)-β1 during the active phase were decreased by injecting MSCs, while the number of mesothelial cells (E-Cadherin) were increased by injecting MSCs. Magnification = ×100. (TIF) [file pone.0043768.s002.tif]

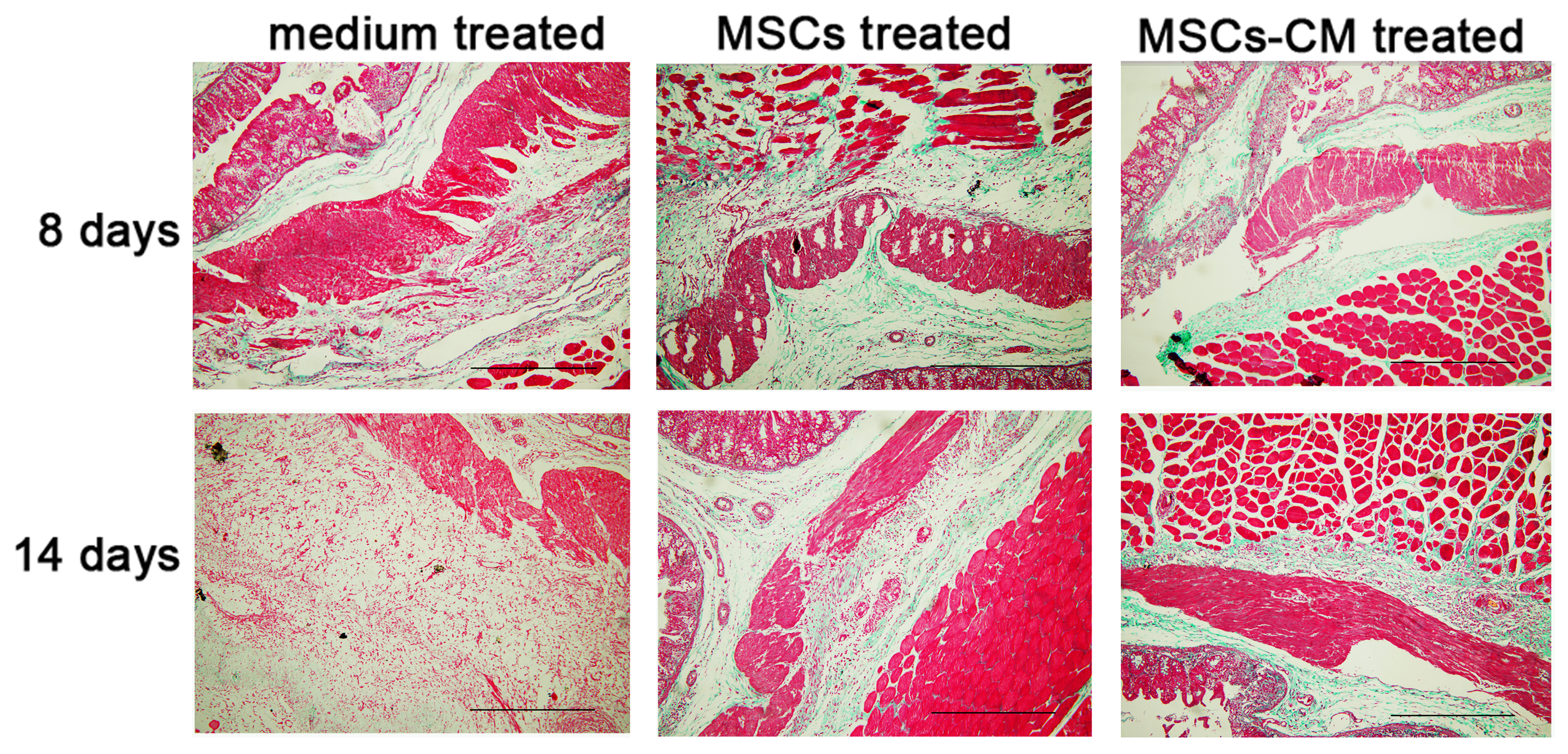

Supplement: Figure S3 — Evaluation of the effects of mesenchymal stem cells (MSCs)-conditioned medium (CM) on acute peritoneal adhesions. Masson's trichrome staining revealed that the fibrosis in the scraped peritoneum was decreased by injecting MSCs-CM. Magnification = ×100. (TIF) [file pone.0043768.s003.tif]

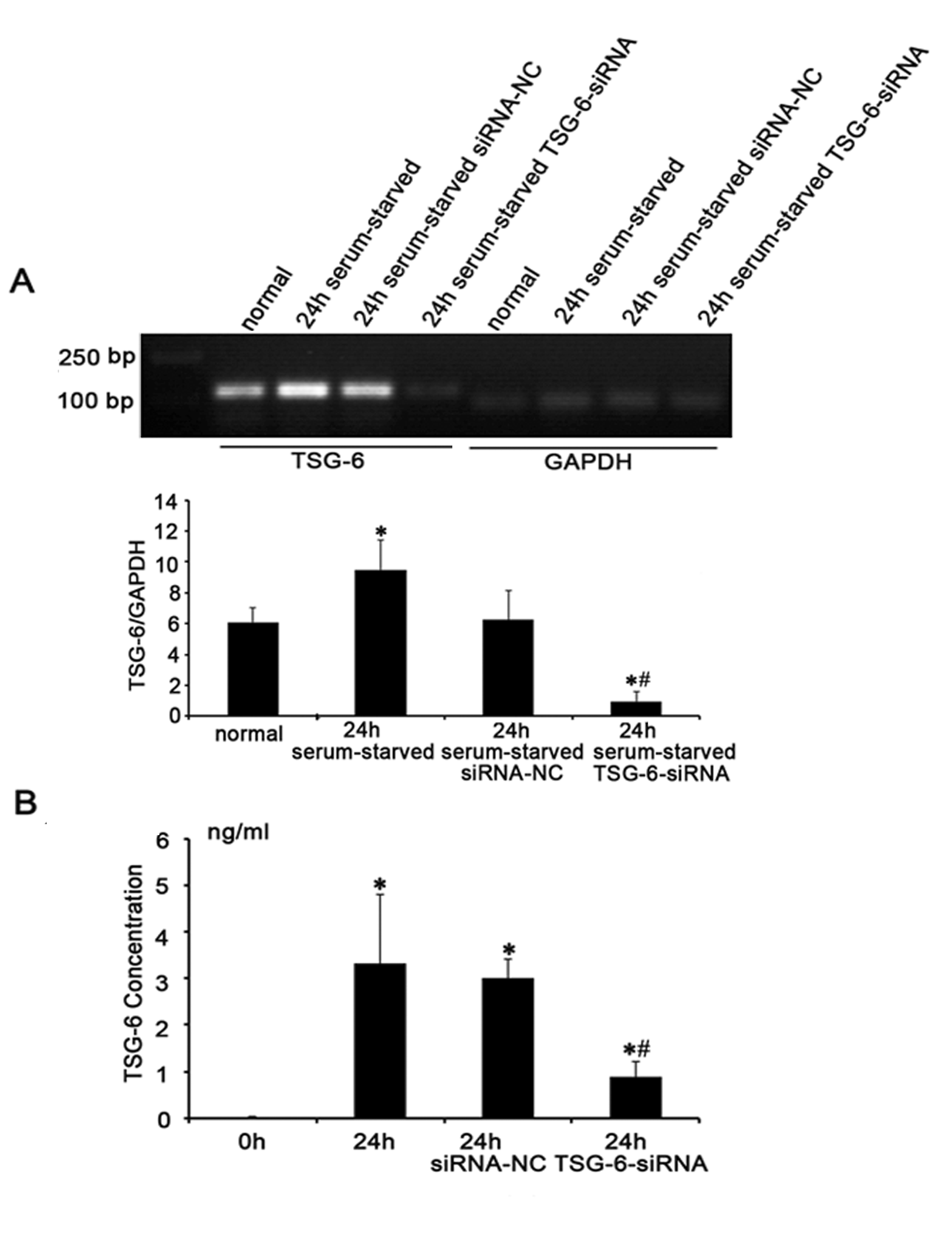

Supplement: Figure S4 — The knockdown efficiency of TNFα-stimulating gene (TSG)-6 in mesenchymal stem cells (MSCs). (A). Knockdown efficiency of mRNA level in MSCs was approximately 82.9% evaluated by reverse-transcriptase polymerase chain reaction (RT-PCR), TSG-6 product length = 134 bp, GAPDH product length = 87 bp. * compared with normal MSCs, p <0.05; # compared with 24 h sreum-starved MSCs, p <0.05. (B). Knockdown efficiency of protein level in MSCs-conditioned medium (CM) was approximately 73.4% evaluated by Enzyme-linked immunosorbent assay (ELISA). * compared with 0h MSCs-CM, p <0.05; # compared with 24 h MSCs-CM, p <0.05. (TIF) [file pone.0043768.s004.tif]

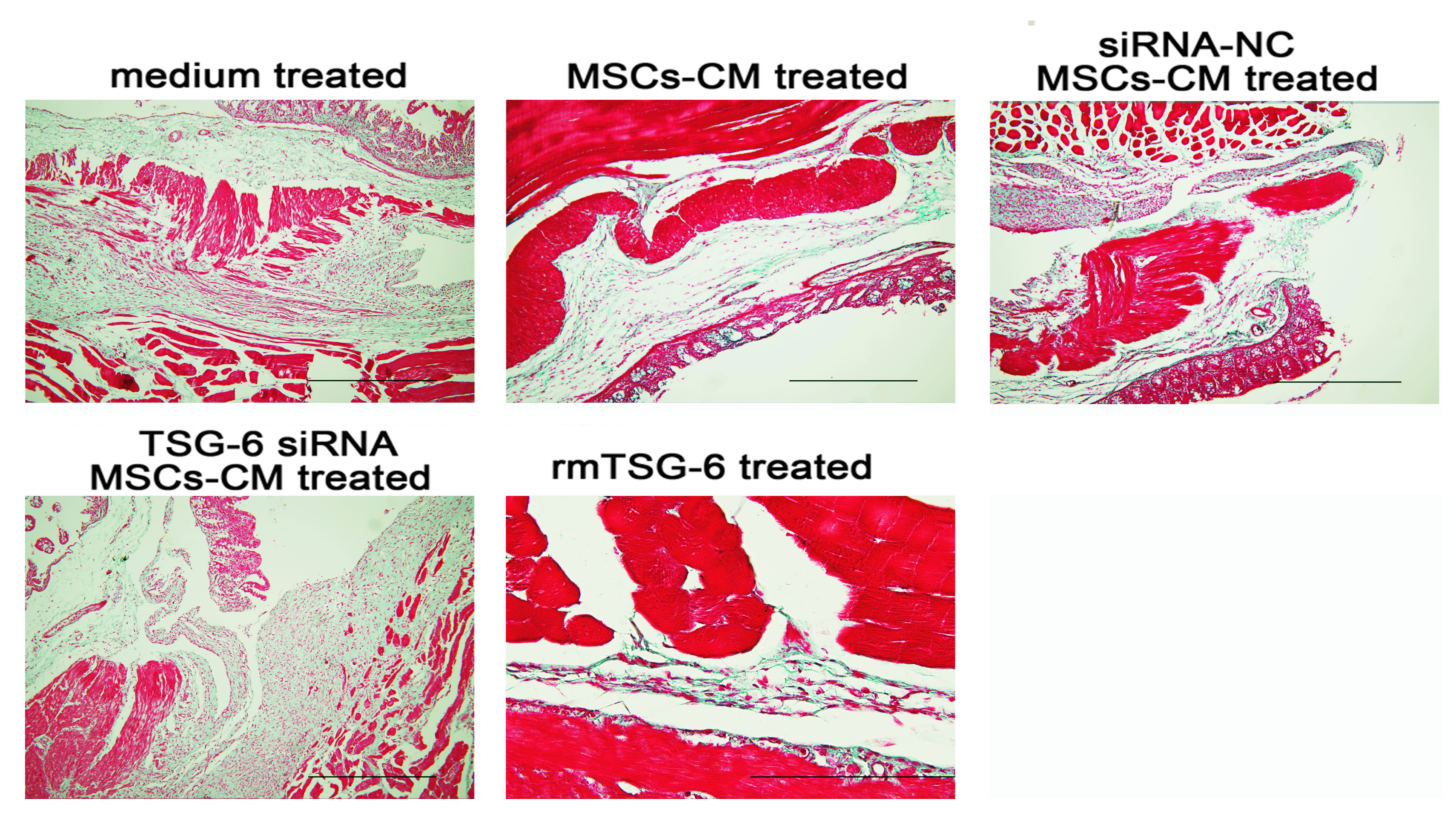

Supplement: Figure S5 — Evaluation of the role of TNF-stimulating gene (TSG)-6 in the reduction of acute peritoneal adhesions by mesenchymal stem cells (MSCs). Histological changes were evaluated using masson's trichrome staining. TSG-6-siRNA MSCs-CM treated group revealed no apparent reduction in the fibrosis of scraped peritoneum. However, the fibrosis was reduced in recombinant mouse (rm) TSG-6 treated group. Magnification = ×100. (TIF) [file pone.0043768.s005.tif]

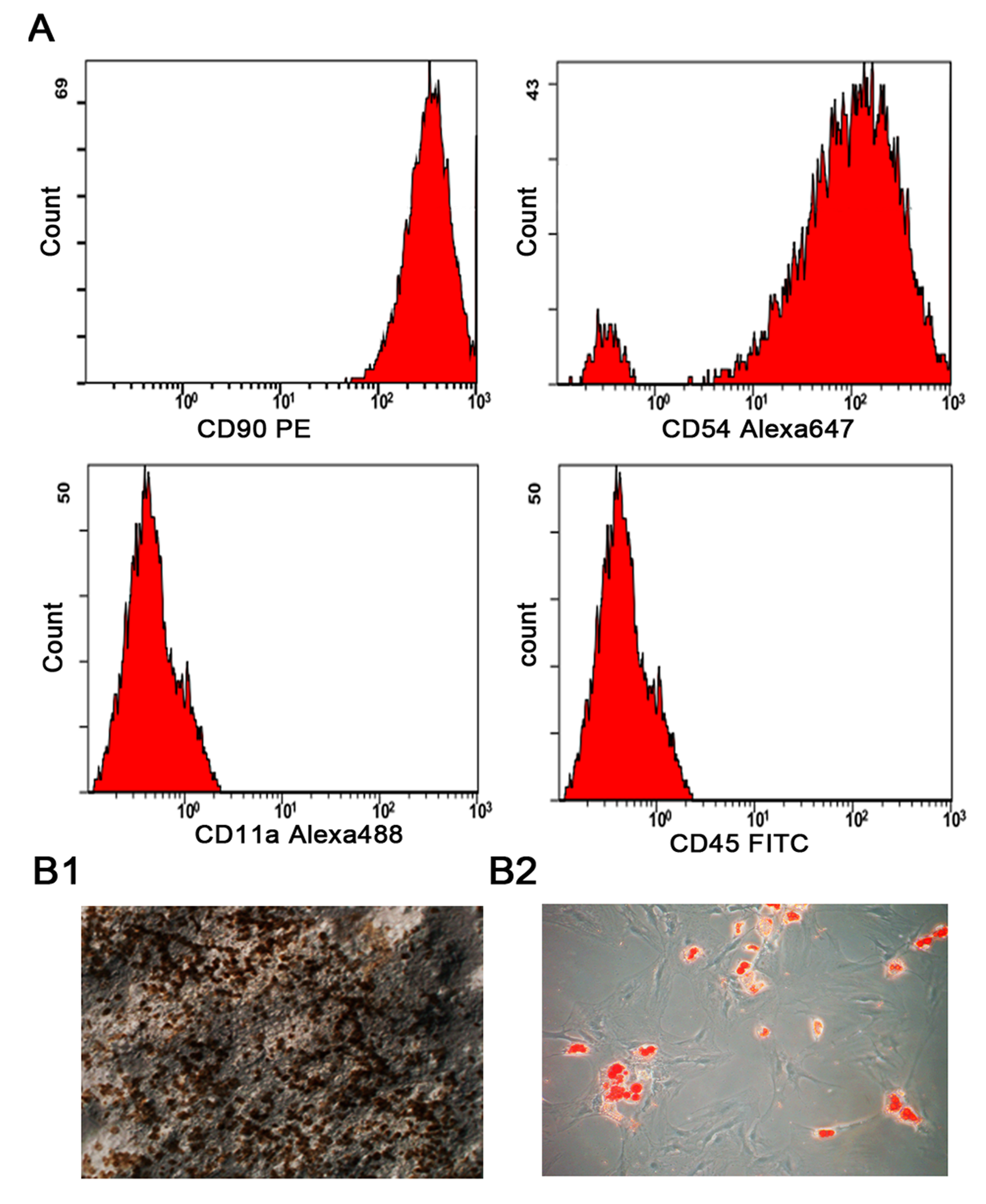

Supplement: Figure S6 — Identification of Sprague-Dawley (SD) rat bone marrow-derived mesenchymal stem cells (MSCs)/green fluorescent protein (GFP). (A). Representative markers characteristic of MSCs. Fluorescence activated cell sorting (FACS) analysis was performed to examine the surface markers of MSCs. The positive proportion of cells displaying CD90 was 97.0%, CD54 was 88.8%, CD11a was 10.7% and CD45 was 8.4%. (B). Multilineage differentiation of MSCs. (B1). Osteogenic differentiation of MSCs. Under osteogenic differentiation conditions, the cells displayed extracellular calcium phosphate precipitates as identified by von Kossa staining. Magnification = ×100. (B2). Adipogenic differentiation of MSCs. Under adipogenic differentiation conditions, the cells accumulated intracellular lipid droplets as revealed by Oil red staining. Magnification = ×400. (TIF) [file pone.0043768.s006.tif]

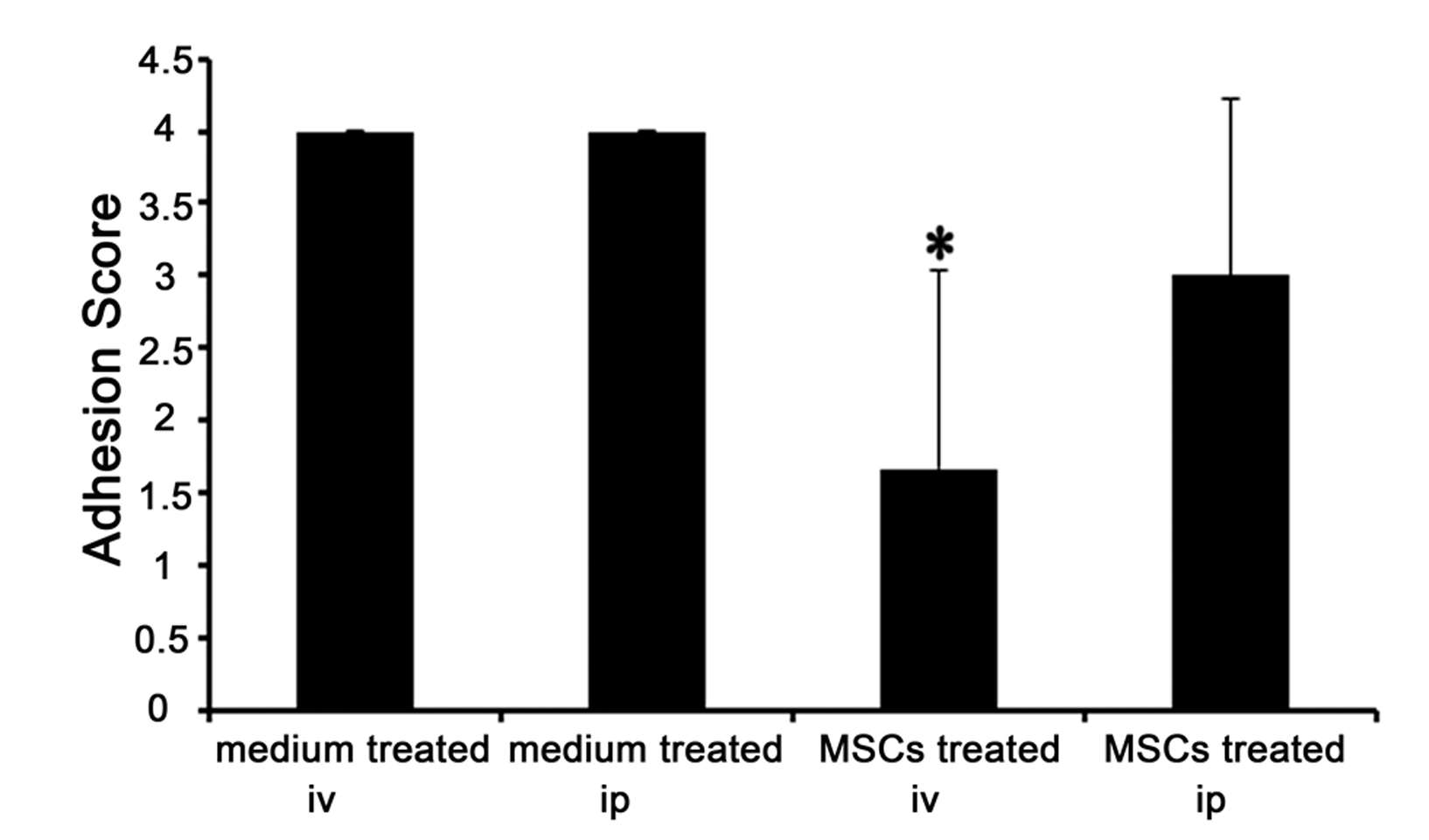

Supplement: Figure S7 — Evaluation of the effects of intraperitoneally injected mesenchymal stem cells (MSCs) on acute peritoneal adhesions. Only MSCs injected intravenously group had lower adhesion scores. The size and severity of peritoneal adhesions were evaluated macroscopically by an independent observer on a scale of 0–4 (0, 0%; 1, <25%; 2, 25–49%; 3, 50–74%; and 4, 75–100% adhesions). * compared with medium treated group (iv), p <0.05, n = 6, respectively. (TIF) [file pone.0043768.s007.tif]
